# Genome-wide distribution of genetic diversity and linkage disequilibrium in a mass-selected population of maritime pine

**DOI:** 10.1186/1471-2164-15-171

**Published:** 2014-03-01

**Authors:** Christophe Plomion, Emilie Chancerel, Jeffrey Endelman, Jean-Baptiste Lamy, Eric Mandrou, Isabelle Lesur, François Ehrenmann, Fikret Isik, Marco CAM Bink, Joost van Heerwaarden, Laurent Bouffier

**Affiliations:** 1 INRA, UMR1202, BIOGECO, Cestas F-33610, France; 2 Univ. Bordeaux, BIOGECO, UMR1202, Talence F-33170, France; 3 Department Horticulture, University of Wisconsin, Madison, WI 53706, USA; 4 HelixVenture, Mérignac F-33700, France; 5 Department of Forestry and Environmental Resources, North Carolina State University, Raleigh, NC, USA; 6 Biometris, Wageningen University and Research Centre, Wageningen NL-6700 AC, Netherlands

**Keywords:** *Pinus pinaster*, Genetic diversity, Linkage disequilibrium, Recombination, Linkage map, Domestication, Breeding program, Forest tree, Genomics, Genomic selection

## Abstract

**Background:**

The accessibility of high-throughput genotyping technologies has contributed greatly to the development of genomic resources in non-model organisms. High-density genotyping arrays have only recently been developed for some economically important species such as conifers. The potential for using genomic technologies in association mapping and breeding depends largely on the genome wide patterns of diversity and linkage disequilibrium in current breeding populations. This study aims to deepen our knowledge regarding these issues in maritime pine, the first species used for reforestation in south western Europe.

**Results:**

Using a new map merging algorithm, we first established a 1,712 cM composite linkage map (comprising 1,838 SNP markers in 12 linkage groups) by bringing together three already available genetic maps. Using rigorous statistical testing based on kernel density estimation and resampling we identified cold and hot spots of recombination. In parallel, 186 unrelated trees of a mass-selected population were genotyped using a 12k-SNP array. A total of 2,600 informative SNPs allowed to describe historical recombination, genetic diversity and genetic structure of this recently domesticated breeding pool that forms the basis of much of the current and future breeding of this species. We observe very low levels of population genetic structure and find no evidence that artificial selection has caused a reduction in genetic diversity. By combining these two pieces of information, we provided the map position of 1,671 SNPs corresponding to 1,192 different loci. This made it possible to analyze the spatial pattern of genetic diversity (*H*_*e*_) and long distance linkage disequilibrium (LD) along the chromosomes. We found no particular pattern in the empirical variogram of *H*_*e*_ across the 12 linkage groups and, as expected for an outcrossing species with large effective population size, we observed an almost complete lack of long distance LD.

**Conclusions:**

These results are a stepping stone for the development of strategies for studies in population genomics, association mapping and genomic prediction in this economical and ecologically important forest tree species.

## Background

Conifers represent an ancient and widespread lineage of about 650 species [1,2]. They are of immense ecological and economic importance as they dominate many terrestrial landscapes and are primarily used for timber and paper production worldwide. Domestication of some of these species started in the mid 1950^ies^ with mass selection of outstanding genotypes in natural forests [3]. Genetic improvement programs resulted in advances in biomass production, wood quality and resistance to biotic and abiotic stresses. However, traditional breeding has remained a slow process because of long generation intervals and because most traits cannot be correctly evaluated at an early developmental stage. The application of genomic techniques in crop [4] and animal [5,6] breeding has resulted in more powerful methods for genetic evaluation, and recent advances in conifer genomics [6-8] have allowed tree breeders to use these tools and methodologies (namely association mapping and genomic prediction) to dissect the genetic basis of phenotypic variability and to accelerate the breeding process of these long-lived organisms [9].

Knowledge about linkage disequilibrium (LD) measured by the squared correlation between two loci is important for applications of molecular markers in association mapping and genomic prediction. The decay of LD over physical and genetic distance determines the resolution and density of the markers required for association mapping [10,11]. A formal link between the power of association tests and LD was established [12], and has recently been generalized for structured populations with related genotypes [13]. LD also determines the accuracy of genomic estimated breeding values [14,15]. Indeed, the direct and inverse relationship between expected LD (r^2^) and population recombination rate (r^2^ = 1/(4N_e_c +1)) has obvious consequences for genomic prediction, because both the training population size and marker density vary with Ne, the effective population size [16,17].

Previous studies of short-distance (physical) LD in conifers, including maritime pine [18,19], have shown that LD extends to only a few hundred to a few thousand base pairs (reviewed in [20]), but with considerable variation between genes [21]. These results have led to the conclusion that millions of SNPs would be required for very high resolution of whole-genome scan association mapping approaches for forest trees,. Thus candidate gene-based approaches have been favored and may prove the best option before sufficiently larger numbers of markers, covering the whole genome, become available [22] as recently illustrated for fruit and forest trees [23], including maritime pine [24]. Considering about 32 thousand genes, with an average gene size of 3–3.5 kb, Pavy et al. [25] estimated that a total of 1.1–1.3 million SNPs would be required to cover the gene space of spruce at a rate of one SNP per 85 bp, which may in any case correspond to only a tiny fraction of the megagenome of this species. Only a few studies have examined the extent and genome-wide distribution of LD in conifers. Using physical information from three random BAC clones, Moritsuka et al. [26] reported significant LD (surprisingly, extending over a distance of 100 kb) in non coding regions of the *Cryptomeria japonica* genome, suggesting that recombination rate may vary according to the nature (coding vs. non coding, low copy vs. repeated sequences) of DNA, as shown in angiosperms [27] and gymnosperms [28]. In the same species, Tsumura et al. [29] discovered that some loci showing divergence along environmental gradients and located in different linkage groups, displayed substantial LD, suggesting an effect of epistatic selection between these loci. To our knowledge, only one study in *Pinus taeda*[30] reported LD for 807 mapped SNPs and confirmed the assumption of independence between genetically linked loci. This study showed that only a handful of loci departed from this expectation, five of which were cosegregating loci displaying a high degree of differentiation between populations. This pattern was attributed to the presence of a ‘genomic island’ of differentiation.

The main objective of this paper was to describe LD pattern, level and structure of genetic diversity across the maritime pine genome. The result may provide baseline information for future genetic studies (association mapping, genomic selection) in this economically important conifer. To this end, we first establish a high-density genetic linkage map by merging three existing SNP-based maps [31] using map merging approaches implemented in the software LPmerge [32,33] and MergeMap [34]. Then, a set of unrelated individuals in the first stage of domestication was genotyped with the mapped markers to describe the genome-wide history of recombination and estimate the level and structure of genetic diversity in this first generation breeding population. Based on knowledge on other forest tree species, we would expect high levels of genetic diversity, a lack of extended LD and limited population structure [22], whereas the applied mass selection might be expected to have decreased diversity around the loci underlying the selected target traits [35]. All of these effects would have important implications for association mapping [36] and genomic prediction in breeding [37].

## Results

### Construction of a composite linkage map for maritime pine and distribution of recombination on chromosomes

**Figure 1:**
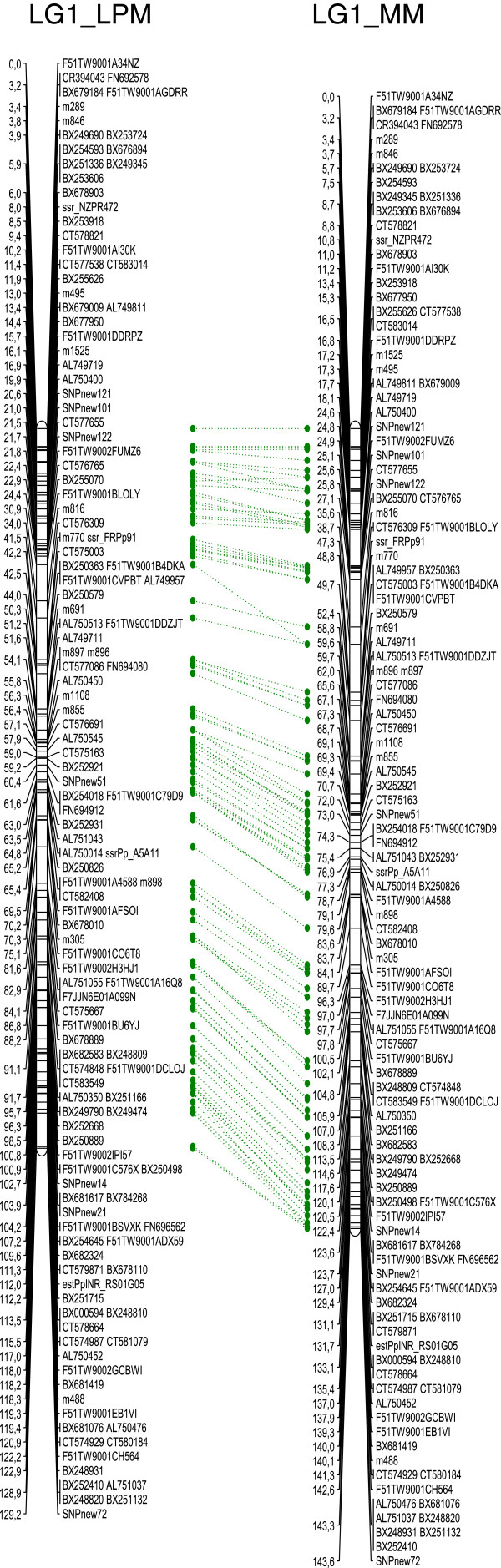
**Alignment of the composite linkage maps (illustrated by LG1) obtained with LPmerge (LPM on the left) and MergeMap (MM on the right) software.** The whole map is available in Additional File 1.

We used the following strategy to integrate the three linkage maps, G2F, G2M and F2, into a single composite map. First, intermediate composite maps were established for G2F-F2 and G2M-F2 because there were few markers common to the G2F and G2M maps suitable for anchoring (25 in total), whereas 198 SNPs were common to F2 and G2F maps and 240 SNPs were common to F2 and G2M maps (see [31]). We then calculated a final composite map from these two intermediate maps. It comprised 1,838 SNPs (1 SNP/contig) distributed along 12 LGs (corresponding to the haploid chromosome number), with a minimum of 121 markers in LG8 and a maximum of 186 markers in LG3. With LPmerge software, the 12 composite LGs covered a distance of 1,712 cM, with individual LG lengths ranging from 115 (LG12) to 182 cM (LG8), and a density of 1 SNP marker per 0.9 cM (Figure 1; Additional file 1). With MergeMap software, the LGs covered 1,850.5 cM, with a individual LG length ranging from 119 (LG12) to 182 cM (LG2) and a density of 1 SNP per cM.

We compared the results generated by LPmerge and MergeMap methods, by carrying out Wilcoxon signed rank tests on two metrics: the linkage group length of the composite map, and the root mean square error (RMSE) calculated from the difference in map position (in cM), between each component map and the resulting composite map. Three hypotheses were tested: i) the map lengths obtained for the intermediate (or final) composite maps do not differ significantly between LPmerge and MergeMap; ii) The difference in RMSE between component (or intermediate composite) maps and the resulting intermediate (or final) composite map does not differ significantly between LPmerge and MergeMap, and iii) the RMSE for each component (or intermediate composite) map does not differ significantly from the intermediate (or final) composite map constructed with LPmerge, and similarly for MergeMap. MergeMap systematically yielded longer maps than LPmerge, for both intermediate and final composite maps (Additional file 2). RMSEs were determined for each linkage group after the map merging process. Comparisons between the two programs showed that MergeMap gave larger RMSEs than LPmerge (optimized for the *K* parameter) for intermediate composite maps, but that RMSEs were similar for the two programs after the final step of map merging (Additional file 2). Despite these differences, marker order was highly correlated (Spearman’s rank R ≥ 0.87, *P* < 0.0001), for all LGs, between the composite maps constructed with LPmerge and MergeMap. Finally, correlations between marker positions on parental maps (F2, G2F and G2M) and on the final composite map constructed with LPmerge were high (Spearman’s rank R ≥ 0.95, *P* < 0.0001), indicating that the positions of the markers on the composite map were consistent with those on the corresponding source maps.

**Figure 2:**
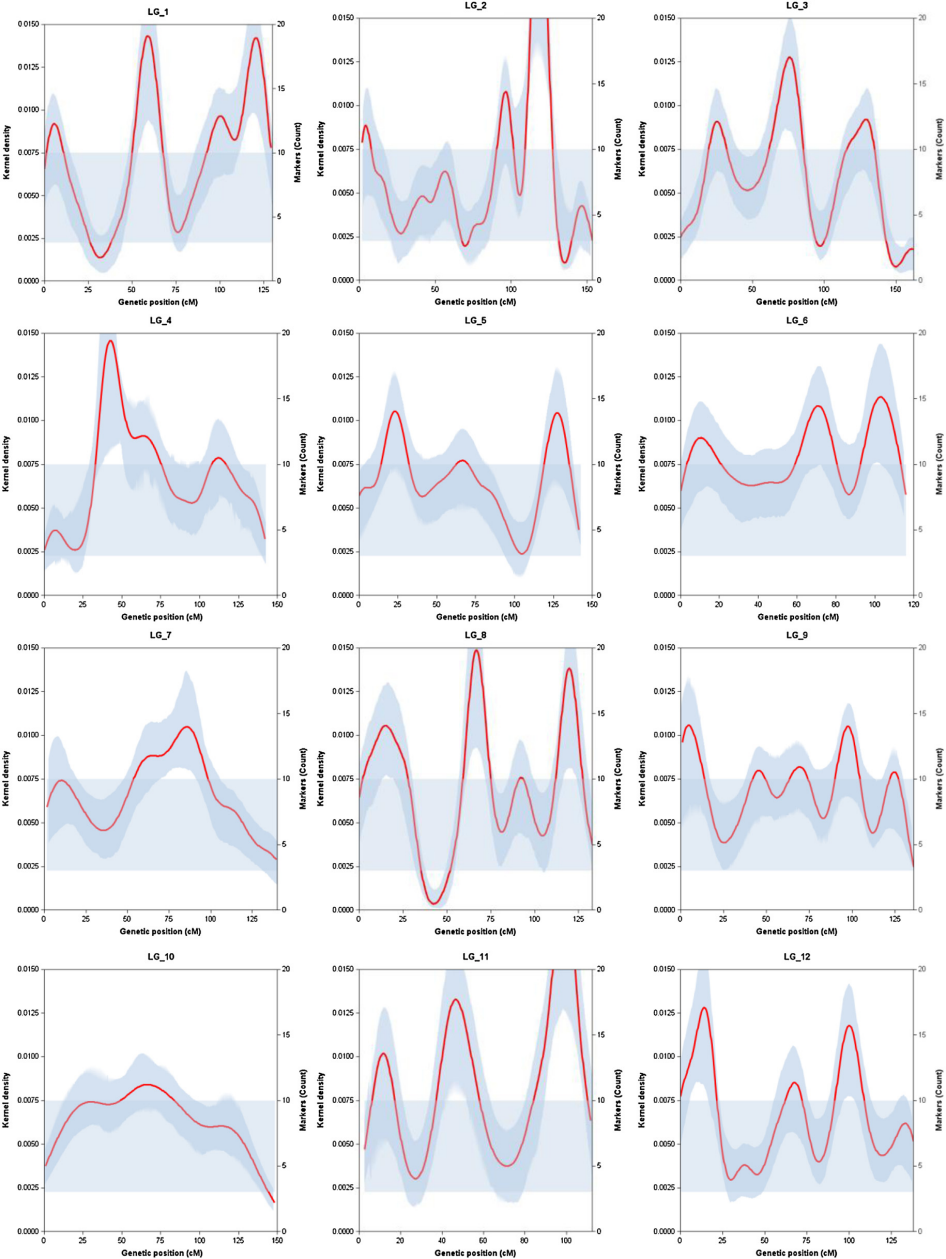
**Kernel density estimators (left y-axis) of marker density (right y-axis) along each linkage group (x-axis in cM).** The red curve corresponds to the kernel density estimator. The surrounding bandplot (in dark blue) is the confidence interval of the kernel density estimator. The horizontal bandplot (in light blue) is the range of variation of marker density under a Poisson distribution. When the lower or upper limit of the confidence interval is above or below this range, we declare the presence of a significant cold or hot spot of recombination, respectively.

A chi^2^-test (df=11) was performed on the composite map, to determine whether genes were evenly distributed between maritime pine chromosomes. With twice as many markers than in our first investigation [31] it was clear that the number of markers per LG did not deviate significantly from a uniform distribution over the 12 linkage groups (*P* = 0.65). In terms of the distribution of markers on individual chromosomes, we found that a density of at least 10 markers per bandwidth (*P *= 3.3 × 10^-63^) was required for the identification of a recombination cold spot, whereas a density of at most three markers (*P *= 3.2 × 10^-63^) characterized a hot spot for recombination. Given these upper and lower limits, and considering the stringent confidence interval defined for kernel density function, we identified 13 significant clusters of markers (in 8 LGs), corresponding to recombination cold spots (Figure 2). It proved more difficult to identify significant hot spots of recombination (we found only two). As reported in [31], hot spots are more genetically variable, and it is therefore more difficult to detect them on a composite map maximizing the number of recombination events from individual crosses. Examination of the shape of the kernel density estimate revealed that seven linkage groups (LG1, 3, 5, 6, 8, 11, 12) had three clear peaks, with locations consistent with the centromeric and telomeric regions. Compared to the study by Chancerel et al. [31] more rigorous statistical testing (using resampling to define confidence interval) certainly contributed to discard a number of false positives. However, one should not forget that the distribution of recombination is genetically variable, therefore by merging information from different genetic maps it is likely that only stable hot and cold spots across the studied genetic backgrounds were revealed.

### SNP-assay genotyping statistics for the first-generation breeding (FGB) population

The mean call rate (percentage of valid genotype calls) was 92% for the FGB population. Two poorly performing samples were identified by plotting the sample call rate against the 10% GeneCall score. Three pairs of trees were found to display identical genotypic information for the 2,600 SNPs and were therefore considered mislabeled in the tree archive (Additional file 3a). All six trees were discarded. This left 186 trees for the analysis of population genetics parameters. In total, 2,600 SNPs were polymorphic (2,532 SNPs and 68 indels), corresponding to 1,706 contigs of the maritime pine unigene (PineContig_v2, [31]). We positioned 1,671 of these SNPs, corresponding to 1,192 different loci, on the composite map. The overall conversion rate (number of polymorphic SNPs or indels divided by the total number of SNPs or indels in the assay, i.e. 9,279 SNPs) was therefore 28%. In total, 2,605 of the 3,498 “failed” assays corresponded to SNPs and 893 to indels, whereas 1,162 of the 3,181 monomorphic loci corresponded to SNPs and 2,019 corresponded to indels. This increased the conversion rate to 40.2% for SNPs and decreased the rate for indels to 2.3%, indicating that indels should be avoided when designing an Infinium assay. A list of polymorphic SNPs is available from the NCBI dbSNP database (http://www.ncbi.nlm.nih.gov/SNP) and is also provided in Additional file 4.

### Test for Hardy–Weinberg equilibrium, distribution of minor allele frequency and population structure analysis

**Figure 3:**
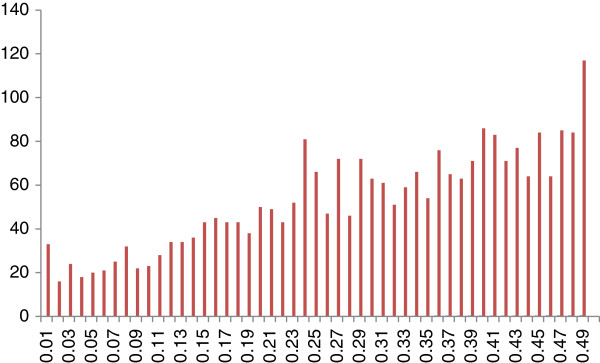
Minimum allele frequency (MAF, x-axis) distribution of each SNP in the first-generation breeding (FGB) population.

Significant departure from Hardy-Weinberg equilibrium was detected for 12 SNPs from the 2,600 polymorphic markers in the FGB population (5% type I nominal error). After Bonferroni correction for multiple tests (5%/2,474 independent tests, although they were not all independent, i.e. an experiment-wise type I error of 0.002%) none of these SNPs yielded a value significantly different from the expected value. We can therefore consider that the percentages of each of the three SNP genotypes remained constant in what can be considered a large population, with random mating, without mutation, migration or natural selection. The minor allele frequency (MAF) distribution of these 2,600 SNPs is shown in Figure 3. A total of 106 SNPs presented a MAF<5%. The scatter plots of these rare SNP alleles were checked visually, one-by-one, with GenomeStudio genotyping software. In all cases, the clustering profile was confirmed. This distribution is unlikely to reflect the true MAF distribution for SNPs in the studied population. Indeed, as pointed out in [31], *in silico* SNP detection based on the use of sequenced cDNA libraries introduces an ascertainment bias toward genes that are strongly expressed (as they are called from expressed sequence tags) and, probably, less polymorphic, due to the stringent cutoffs used: i) MAF≥33% and coverage≥10×, to prevent the selection of SNPs present at such low frequencies that they are likely to be the product of sequencing error, ii) ADT score ≥ 0.75, to minimize the variability of the flanking region surrounding the targeted SNP, thereby increasing the likelihood of a successful Illumina Infinium assay. In addition, the MAF spectrum is likely to be shifted upward, with an underrepresentation of rare alleles not captured due to the small size of the sample used to prepare the cDNA libraries. The possibility of such a bias should be borne in mind when making further evolutionary inferences concerning the demographic and selective history of maritime pine populations.

Population structure and relatedness between individuals are known to bias the estimation of LD [13]. In this study, the trees of the FGB population were selected from natural stands in the Landes forest, with a sampling method designed to ensure the sampling of unrelated individuals. The observed patterns of pairwise relatedness (Additional file 3b) suggests that this objective was achieved. We tested for possible cryptic relatedness or differentiation, by performing principal component analysis (PCA) on the full genotype matrix of 2,600 SNPs. A comparison of the size of the eigenvalues obtained with the Tracy-Widom distribution yielded two significant principal components. In theory, this could indicate the presence of three distinct subpopulations, clustering on the basis of the first two PCs yielded three groups with very low levels of genetic differentiation (Fst 0.002-0.005). We plotted these individuals along the two significant PCs and found little evidence of separate clusters (Additional file 5a). Geographic analysis reveals a significant relationship (r^2^ = 0.17, *p *= 0.007) between genetic PC1 and the major axis of geographic variation (mostly latitude), with some evidence of PC2 being associated with the second axis (mostly longitude) (r^2^ = 0.06, *p *= 0.11) (Additional file 5b,c,d). Overall, there was a weak, but significant pattern of isolation by distance (r = 0.2, *p* = 0.006) (Additional file 5e) rather than a division into distinct groups. This result was confirmed by the structure analysis performed with Structure software (Additional file 6). In this analysis, the values of mean likelihood obtained for the one- to ten-group models tested did not reach a plateau and Evanno’s delta K criterion did not identify a peak for any of the K values tested. Moreover, for K values ranging from 2 to 10, the entire set of 186 individuals was found to be admixed, with none being identified as a full member of a specific group. These patterns are typical of an unstructured population [38] and indicate the absence of a particular genetic structure at the scale of the FGB population.

### Spatial analysis of genetic diversity on chromosomes

The mean value of Nei’s diversity index (H_e_) calculated for the 2,600 SNPs was 0.391 (SD = 0.127), while that for the 1,421 SNPs corresponding to mapped contigs was 0.434, (SD = 0.067). These are very high estimates given the biallelic nature of these markers (the maximum H_e_ being 0.5 for a biallelic marker). We used the mapped markers to determine whether genetic diversity was equally distributed between the LGs (i.e. presence of LGs with lower or higher overall diversity, Additional file 7). A significant difference (*P*<0.05) between H_e_ values was observed. Tukey’s HSD test showed that LGs could be classified into three groups, with lower (LG3-6, H_e_ = 0.419; SD = 0.072), medium (LG1-2-7-8-9-10-11-12, H_e_ = 0.434; SD = 0.066) and higher (LG 4–5, H_e_ = 0.449; SD = 0.059) levels of diversity.

**Figure 4:**
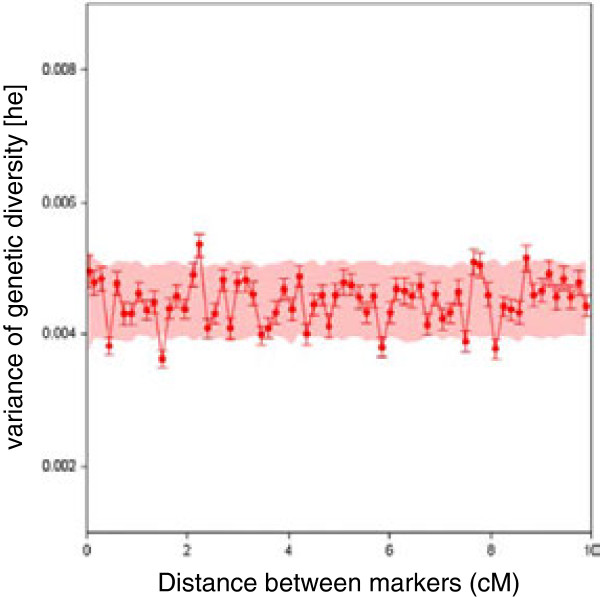
**Empirical pangenomic variogram of genetic diversity (H**_**e**_**) plotted against map distance, ranging from 0 to 10 cM (0.15 cM as the lag size).** The red dots (connected by a red line for trend detection) represent the variance of H_e_ for each pair of markers for a given class of map distance. The bandplot indicates the 95% confidence interval.

We then used a spatial statistics approach to determine whether the genetic diversity of the mapped markers was distributed non-uniformly along the chromosomes. We estimated the empirical variogram of *H*_*e*_ (${\hat{\gamma }}_{h}$), to determine whether neighboring genes on the chromosome presented similar patterns of diversity. A spatially structured process would show an increase in variance with increasing map distance between markers. Based on all the gene loci from the composite map and map distances ranging from 0 to 10 cM, we found no particular relationship between ${\hat{\gamma }}_{h}$ and gene position on the composite map. Most of the calculated empirical variances fell within the area predicted by permutation (Figure 4). This was true for the individual LGs of the composite map and was confirmed for the component maps as well (data not shown). Thus, diversity at neighboring gene loci was not correlated with recombination distances in the study population and, with the marker density used, there is little evidence for extended reductions in diversity due to selective sweeps. Given this result, we did not attempt to krige our data to detect hot or cold spots of diversity at a centimorgan scale.

### Extent of intra- and inter-chromosomal linkage disequilibrium

At least two SNPs were available in 248 EST-contigs for investigation of the pattern of physical LD. We considered SNPs with a MAF>5%, resulting in the retention of 714 pairs for the analysis. However, given the biased procedure used to select SNPs *in silico*, the biased representation of polymorphic sites within these contigs and the skewed distribution of distances between sites (half the pairs being at a distance < 250 bp), the observed pattern of short-distance LD (not shown) was not consistent with trends typically observed in conifers [39] based on amplicon sequencing. In addition, the estimate of the population experimental parameter (C) was negative, precluding any use of this data set for the further interpretation of physical LD over short distances.

**Figure 5:**
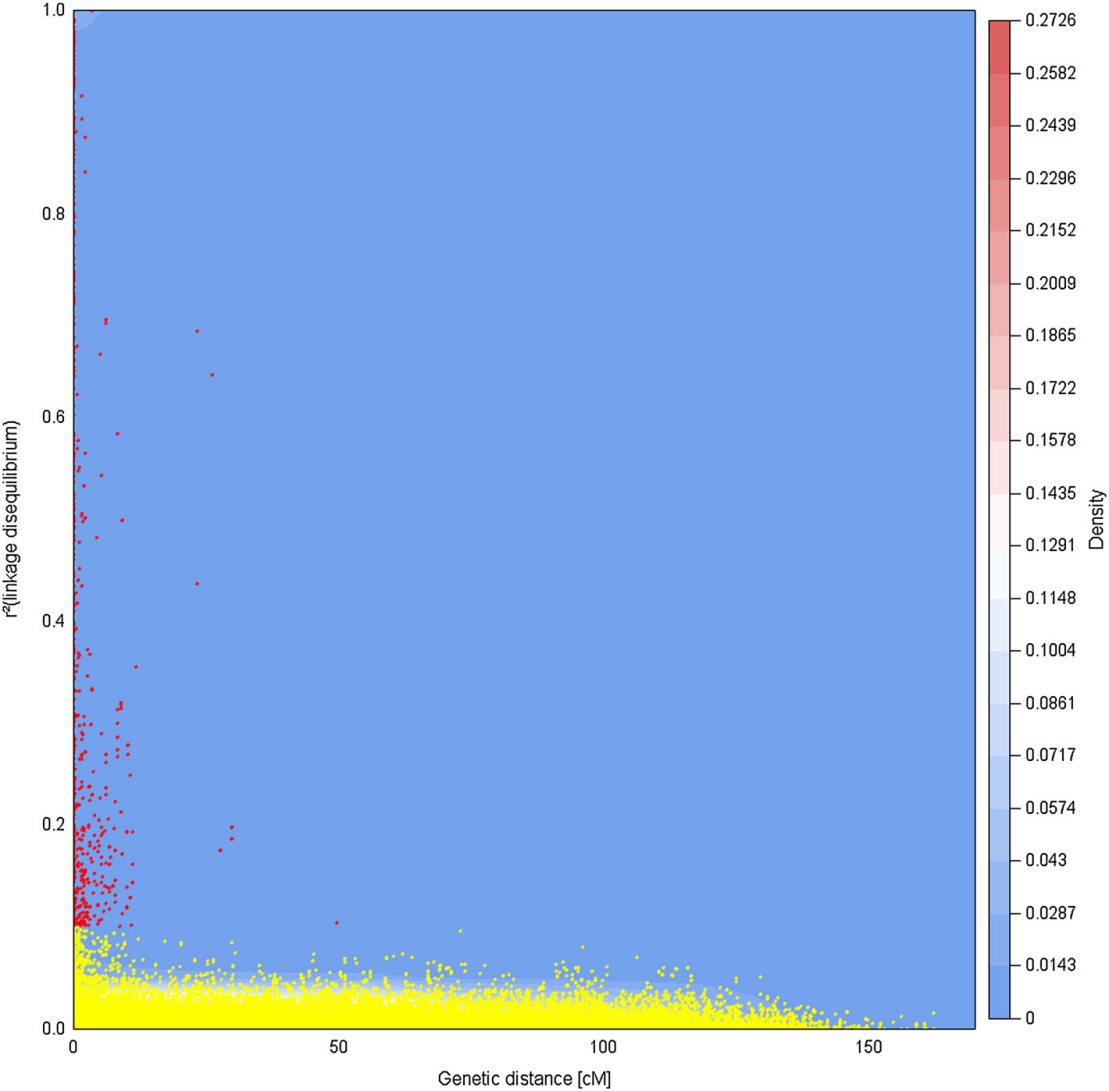
**Distribution of intra-chromosomal linkage disequilibrium (r**^**2**^**) as a function of physical and genetic distance between all marker pairs for the 12 linkage groups of the maritime pine composite linkage map (see Additional File****5****, for each LG independently).** SNPs from the same contig were placed at 0 cM. r^2^ was determined by the Rogers and Huff [93] approximation for loci with unknown phase, based on the polymorphic data for 186 unrelated trees of the Aquitaine population. In yellow: r^2^ values below a cutoff value of 0.1. In red: r^2^ values above this cutoff.

**Figure 6:**
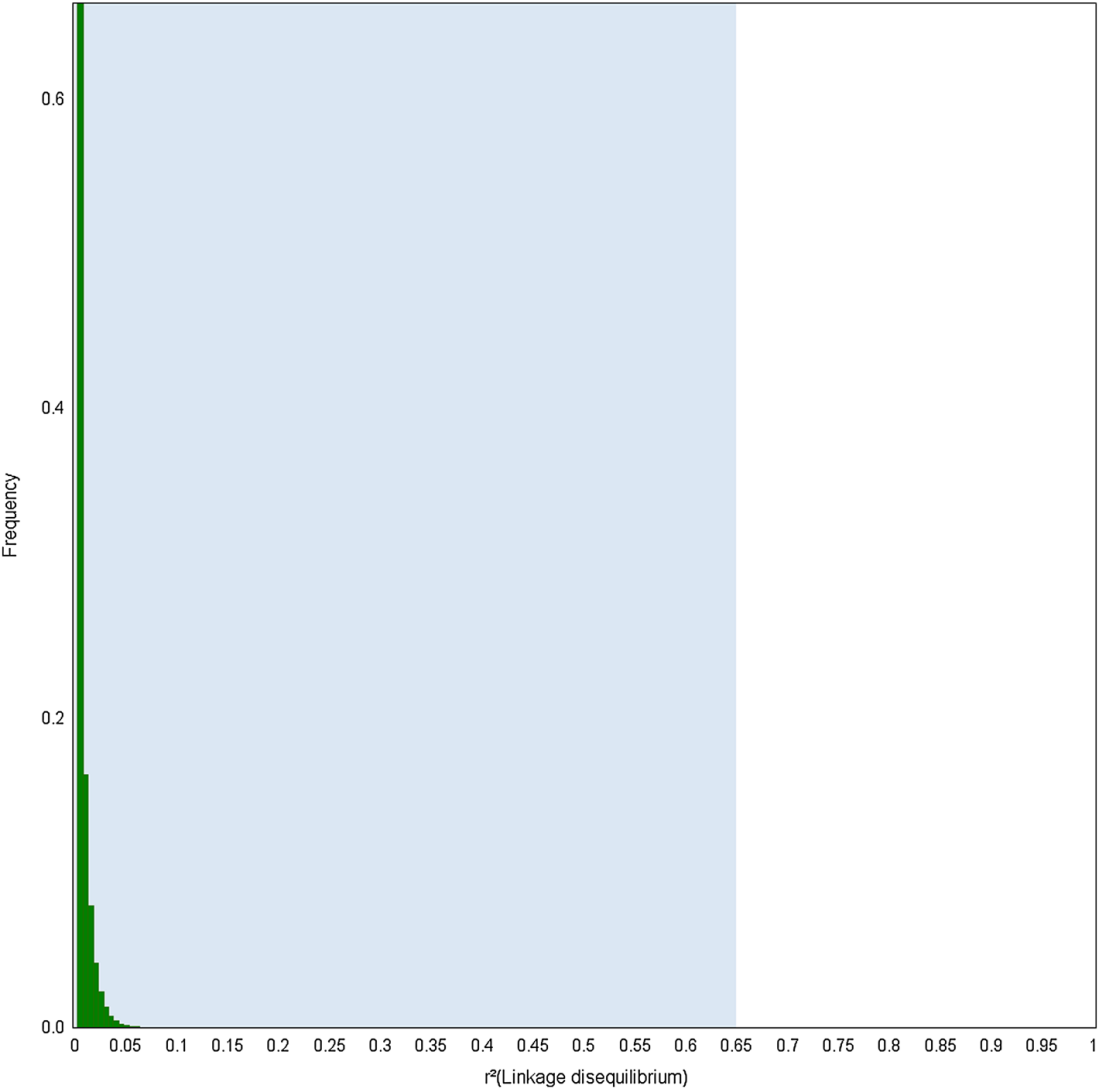
**Distributions of inter-chromosomal LD (green bars).** The blue area corresponds to expected values under a null model obtained for non physical LD. The upper bound value of this blue area was corrected for multiple testing (Bonferroni correction, *P*-value=0.05/159,814).

The pattern of long-distance (genetic) LD was examined for the first time in this species, over the 12 chromosomes, on the basis of SNP markers localized on the composite linkage map and their genotypic profiles in an unstructured population. The distribution of the squared correlation coefficient for allelic frequencies (r^2^) showed that LD decreased rapidly over very short genetic distances for all chromosomes (Figure 5; Additional file 8). However, we also identified 380 pairs (0.45% of the 84,679 pair-wise combinations) for which the r^2^ was above the 0.1 critical level, while the genetic distance was different from 0 in the composite map. In order to verify whether these possible long distance LD (possibly due to epistatic selection) were not due to inaccurate map position resulting from the construction of the composite linkage map, we directly checked the map position of these pairs in the components maps. From these 380 pairs, 238 originated from the same component map, while 142 were from different component maps. From these 238 pairs, the genetic distance in the component map was equal to 0 cM for 102 pairs and comprised between 0 and 1 cM for 66 pairs, indicating that their position in the r^2^ plot was probably unreliable and therefore could not be used to infer long distance LD. An extreme case is provided for two outliers markers (F51TW9001DHGV3 and CT583376) in LG3 placed 23 cM apart in the composite map, while they completely co-segregated in the component map (G2M). Thus, only 70 pairs (i.e. 238–102–66) were left to construct the distribution of long distance (i.e. non physical) LD. As rare allele frequency can influence LD, this distribution (Additional file 9) was drawn based on 65 pairs (listed in Additional file 10) from which both markers had a MAF >20%. In cases where a functional annotation was available, there was no similarity between a marker pair suggesting that these SNPs belonged to different genes rather than to different contigs of the same gene. In addition, 34 cases (highlighted in bold in Additional file 10) of such possible long distance LD could be confirmed by the fact that intragenic SNPs presented similar r^2^ values with SNPs in another gene. Finally, this distribution was used as a null model to test the significance of inter-chromosomal LD (potentially due epistatic selection). Each inter-chromosomal LD value was tested against the upper bound of this null distribution (significant if r^2^ > 0.32 at the 5% level). Given the number of tests performed, Bonferroni correction was applied to this upper bound (see the blue area in Figure 6). No significant inter-chromosomal LD was found in this population.

## Discussion

### Development of a composite map for maritime pine and genome-wide distribution of recombination

Advances in next-generation sequencing and array-based genotyping technologies have lowered development times and costs for reliable single-nucleotide polymorphism (SNP) markers [40,41]. The availability of such markers has been a boon for the generation of high-density linkage maps in model and non model plant species, as recently demonstrated in sunflower [42], barley [43], tomato [44], and maize [45]. The integration of information from multiple linkage maps for hundreds to thousands of markers is another challenge. One approach to the integration of information for multiple populations is to pool the genotypic data and minimize the sum of recombination frequencies (or related metrics), as in the maximum likelihood method [46] applied to single populations, e.g. [47]. However, the computational time required for this approach may be prohibitive in some situations [48,49] and this method is unworkable when genotypic data are unavailable. An alternative strategy involves integrating the linkage maps for separate populations without analyzing their genotypic data. Yap *et al.*[50] were the first to model a map as a directed graph, with nodes representing mapped markers and edges defining the order of adjacent markers. They also designed an algorithm for merging maps from different studies on the basis of loci common to different maps. Wu *et al.*[34] subsequently developed an algorithm based on graph theory implemented in MergeMap, a program that has been used to construct several composite maps for barley [43,51]. Endelman [32] discovered that the graph linearization technique used by MergeMap was suboptimal and proposed a new approach to overcome this problem through linear programming. However, the software developed by Endelman [32], DAGGER, was unable to merge linkage maps with ordering conflicts. LPmerge, used for the first time on empirical data in the present paper, was designed to resolve ordering conflicts between component linkage maps and minimize errors between the composite map and the component maps [33]. By using this software we generated a composite map consisting of 1,838 SNP markers distributed over 12 LGs, covering 1,712 cM. Map length was similar to that obtained for maps constructed with similar numbers of loci in other conifer species: 2,083 cM in *Picea glauca* with 1,801 loci [25], 1,898cM with 1,816 loci in *Pinus taeda*[30]. We then used this map to investigate the genome-wide distribution of recombination. We found clear peaks for the number of markers. Their locations was consistent with centromeric and telomeric regions, in agreement with previous findings in other species with a similar genome size such wheat, reporting that recombination was limited in these regions [52,53].

### Level and genome-wide distribution of genetic diversity in the first breeding population of maritime pine

We presented a genome-wide map of genetic diversity (as estimated from expected heterozygosity, H_e_) for a population resulting from mass selection in natural forests, with an estimated selection intensity of about 1.5 × 10^-5^[54]. This population provided us a unique opportunity to study the effect of the first stage of domestication on the level and distribution of genetic diversity in a highly heterozygous forest tree species. We showed that a selection intensity of this magnitude did not decrease the overall level of genetic diversity.

Our findings are consistent with those of previous studies carried out with an handful of allozyme markers in breeding populations of Douglas fir [55] and Sitka spruce [56], and with a recent investigation based on SNP markers spanning the entire genetic map of white spruce [57], showing no decrease in genetic variation during the first stage of domestication of these highly polymorphic species. We can therefore conclude that mass selection applied at a regional scale (the Landes forest covers about 1 million ha in the southwestern France), even with very high intensity, did not appear to compromise the background neutral genetic diversity of the maritime pine base breeding population. Thus, the high level of genetic diversity found in the FGB population is consistent with a large randomly mating population, as typically found for outcrossing species.

We found no significant spatial pattern of genetic diversity in the maritime pine genome (at least at the cM scale). Such patterns would have been indicative of decreases in diversity associated with loci underlying the variation of the target traits. However, given the rapid decay of LD in this species (within a few hundred bp on average), the marker density used was probably too low to capture any localized decline in heterozygosity, if any occurred around selected loci.

These results contrast with the large reduction of genetic variability observed for the selected traits [58] between the Landes natural forest and the base population of the breeding program (which includes the FGB population), particularly for growth. We can therefore conclude that these markers are probably not functionally important with respect to these selection criteria, in agreement with the lack of statistical association between allelic variation and breeding values for height growth and stem straightness (data not shown). Further investigations will be required to identify SNPs in LD with target trait-QTLs. Such investigations could involve the genotyping of unselected trees from wild populations and the comparison of allele frequencies before and after mass selection, or tests of association between breeding values and marker genotypes, as illustrated in [57] for white spruce. Given the polygenic basis of complex traits subjected to breeding, such as height and radial growth [59], we anticipate that this second approach is likely to be successful only for well chosen candidate genes putatively involved in trait variation.

The set of 2,600 SNP markers developed in this study will be used to assess genetic diversity in subsequent generations of the maritime pine breeding program. The maintenance of genetic diversity is not only essential to guarantee the adaptation of future improved varieties to ongoing climatic change [60], it is also of particular importance for plant breeding programs based on recurrent selection, because the progress of selection is determined by the level of genetic variation within the population.

### Long distance LD pattern and consequences for association mapping and genomic prediction in maritime pine

We scored 2,600 SNPs in a population of 186 unrelated trees selected on the basis of their performance in natural forests of the Aquitaine region in southwestern France, for establishment of the first generation of the maritime pine breeding program. Markers for which intra-chromosomal LD was estimated covered the whole linkage map of this species, at a mean density of 1 marker per 1.4 cM (1 cM ≈ 12 Mb in maritime pine, [61]). Sampled genes were well distributed across the 12 LGs of the composite map, with 78–115 genes per LG. As expected, high values of r^2^ were obtained only for physically linked polymorphisms, i.e. SNPs belonging to the same gene. No significant LD was found over larger distances. These results are consistent with population genetics theory for such an undomesticated, outcrossing species, and can be attributed principally to the large effective sizes of the unstructured populations found in most conifers (estimates of effective population sizes for maritime pine are presented in Additional file 11). Similarly, no significant epistatic LD was found between unlinked loci localized on different chromosomes. LD is a property of a given gene pool, but the convergence of our results with those of Eckert *et al.*[62] for *Pinus taeda* suggests a lack of LD between genetically spaced gene-based markers in conifer species characterized by the same type of reproductive regime and life history traits.

Our findings suggest that the initial mass selection used to form the base population of the maritime pine breeding program was not only successful in terms of the initiation of a program to develop improved varieties [58], but also efficient for the sampling of neutral genetic diversity from the Landes forest. Absence of inbreeding and cryptic population structure within the base population were also confirmed. The substantial level of polymorphism detected in the FGB population renders our set of markers as a valuable tool for breeding applications. Trees have long generation interval and breeding is therefore a slow process. The 2,600 SNPs developed in this study will be extended to test the utility of genomic selection (GS) approaches to reduce the breeding cycles of maritime pine, as suggested for *Pinus taeda*[63,64]. Then, favorable combinations of polymorphisms will be sought in manageable breeding populations with small effective sizes to trace QTLs by linked markers. The prospective of developing GS holds great promise to increase the genetic gain in traits of interest in these long-lived organisms and to accelerate their domestication [65,66], while maintaining sufficiently high levels of genetic diversity to allow the selected trees to cope with major biotic and abiotic disturbances.

Given the lack of LD in this population and lack of associations between markers and phenotypes, predictions based on SNP markers for selection would likely have very low reliability. In several simulation studies on domestic animal and trees, LD showed a significant effect on reliability of predictions from genomic prediction models [15,16]. For example, in cattle breeding, for genomic selection to be successful the level of LD was suggested to be greater than 0.2 [14]. When LD among the markers increased from 0.1 to 0.2, the reliability of genomic predictions increased by 0.14 (from 0.68 to 0.82) [67]. LD is population specific and is expected to change with recombination, genetic background of the population and effective population size. To exploit marker-tagged QTL-trait associations in GS, we are currently combining three-generation pedigrees of maritime pine (FGB and successive G1 and G2 populations), where LD should be much higher compared to the base population.

## Conclusions

We established a 1,712 cM linkage map of maritime pine with 1,838 SNP markers using for the first time a new map merging algorithm that integrates linkage maps from separate populations without any recourse to original genotypic data. We found clear cold spots of recombination consistent with the centromeric and telomeric regions of metacentric chromosomes [68]. We then used an extended set of 2,600 SNP markers to describe historical recombination, genetic diversity and genetic structure within a mass-selected population of 186 unrelated genotypes. The genetic structure of this population was very weak and we found no evidence that artificial selection had decreased neutral genetic diversity. Considering the map position of 1,671 of these 2,600 markers (corresponding to 1,192 different loci) we found that LD mostly extend over short physical distances as expected for an outcrossing species with large effective population size.

At the dawn of a new paradigm in forest tree breeding [69-71], namely the implementation of GS [37], a range of factors that influences the accuracy of genomic estimated breeding values needs to be carefully considered, including the heritability of the traits, its genetic architecture, the extent of genotype by environment interaction, the genetic structure and the effective size of the targeted population, the number of records in the reference population, the number of markers and their associated cost, and the overall prediction and validation strategy. The present study provides novel results that should be taken into account for the implementation of GS in maritime pine. The drop in the status number (as defined by [72]) from several hundred in the mass-selected population, to 94 in the second breeding population and 23 in the elite population of the new sub-line structure of the breeding population (A. Raffin personal communication) is a favorable situation for its further development in this species.

## Methods

### Genetic material, DNA extraction and genotyping assay

The two mapping populations (G2 and F2) for which SNP-based linkage maps were merged in this study were described in [73]: G2 designates a three-generation outbred pedigree (full-sib progeny), whereas F2 is a three-generation inbred pedigree. Chancerel *et al.*[31] constructed male and female linkage maps from the G2 population (G2M and G2F, respectively), and a single linkage map for the F2 population (Additional file 12). In addition, 194 trees from the base population of the maritime pine breeding program, referred to here as the “first-generation breeding” or “FGB” population, were used for genetic diversity and LD analysis. During the 1960s, adult stands in the Landes forest (south western France) were explored and trees considered outstanding in terms of their stem volume and straightness were identified. These trees were sampled across a wide range of different locations covering the Aquitaine region (Additional file 13), particularly along the Atlantic coast, and were at least 50 m apart when present at the same site. A phenotypic index was built from the performances of the candidate trees and their 20 closest neighbors [54], to select the base population. These trees were grafted and stored in clonal archives [58].

Young needles from each tree were harvested and stored at -80°C until DNA extraction and genotyping (Infinium assay, Illumina), as described in [31]**.** In total, 9,279 SNPs (6,307 SNPs *sensus stricto* and 2,972 indels distributed in 4,613 different contigs) were individually inspected with Genome Studio software, using a GenCall score cutoff of 0.15 (according to Illumina’s recommendations) to detect failed SNPs. Loci for which two or three clusters (depending on the type of marker segregation) were identified without ambiguity were considered to be polymorphic markers. SNP clusters were modified manually, to refine cluster positions, when necessary. SNPs and surrounding sequences were submitted to dbSNP (accession numbers are listed in Additional file 4). Overall, 186 out of the initial set of 194 trees presented genotyping information for 2,600 SNPs (Additional file 14).

### Linkage map development

We compared two different software packages to generate a composite map from three existing SNP-based linkage maps (G2F, G2M, F2, [31]) of maritime pine: LPmerge [32,33], which is available as an R package [74] at http://cran.r-project.org/web/packages/LPmerge/, and MergeMap (http://www.mergemap.org/), which has been used in several barley mapping projects [34,51]. To compare both algorithms, the root-mean-squared error (RMSE) for each marker was calculated by comparing its position in the composite map with that on the individual linkage maps, and the average RMSE across the markers within a linkage group was used to assess the goodness-of-fit for the composite map. For LPmerge, the maximum interval parameter *K* was varied from 1 to 8, and the composite map with the lowest RMSE was selected. For both software packages, as few markers were common to G2F and G2M, we first generated two intermediate composite maps (“F2+G2F” and “F2+G2M”). We then merged intermediate maps into a final composite map. The merging of the three maps in a single step yielded the same marker order in the composite map (Spearman’s rank R > 0.99, *p *= 2.2.10^-16^, data not shown), but we present the two-step procedure here because this approach made it possible to compare LPmerge and MergeMap on three datasets (“F2+G2F”, “F2+G2M” and the combination of the two), making it possible to draw more general conclusions.

### Analysis of marker distribution on chromosomes

We investigated whether the mapped genes were evenly distributed between linkage groups (LG), by comparing the observed and expected numbers of genes per linkage group in chi^2^ tests (P<0.05). The expected number of genes for each LG was obtained by multiplying the ratio “size of LG/total genome length” by the total number of mapped markers. We also analyzed the distribution of markers along the chromosomes, by using a kernel density estimation to calculate optimal window size (bandwidth) for dividing the genome into blocks, in which we counted the number of genes. Kernel density estimation is a non-parametric technique for density estimation, in which a known density function (here a Gaussian function) is averaged across the observed data points to create a smooth approximation. The smoothness of the density approximation depends on the bandwidth. In our case, we used a fixed and robust bandwidth estimator [75], based on the algorithm of Jones *et al.*[76]. Bandwidth values were calculated for each linkage group of the composite map obtained with LPmerge (Additional file 15). Compared to our first investigation based on the three component maps [31], we estimated here the variability of the kernel density estimator, by sampling randomly 70% of the total number of markers for each chromosome independently, 999 times without replacement [77,78]. For each random sample, we calculated a kernel density estimate. For all the kernel density estimates (from 999 random samples), we then calculated both the 2.5 and 97.5 percentiles, to define the confidence interval of the kernel density estimate. We defined the lower and upper bound thresholds of significance, by analyzing the marker distribution, by comparing (in a chi^2^-test) the observed distribution of the number of markers per bandwidth with that expected under a Poisson distribution. A lower bound threshold, defining a cold spot of recombination (i.e. a cluster of markers on the linkage map) was determined when the observed number of markers was greater than the expected value, while the results of the chi^2^-test were significant. Similarly, to define a hot spot of recombination, an upper bound threshold was determined when the observed number of markers was lower than expected, while the results of the chi^2^-test were significant. Finally, we compared the position of the confidence interval of the kernel density estimator with these lower and upper bounds, to identify significant hot and cold spots, respectively.

### Population structure analysis

Genetic structure and cryptic relatedness within the FGB population were assessed in three ways. First, we assessed the patterns of pairwise relatedness, calculated from the genotype matrix as described in [79]. Second, we tested for cryptic population structure by performing principal component analysis (PCA) on the genotypic matrix of 2,600 markers, as described in [80], removing the dependence between SNPs at the same locus [81]. The leading eigenvalues obtained by PCA were tested for significance, by comparing their size with that expected under a Tracy-Widom distribution [80,82]. Genetic clusters were created on the basis of Ward clustering of the calculated Euclidean distance from the significant PCs [81]. Significant PCs were averaged per geographic location (sampling site) and their relationship to geographic location was investigated by linear regression on the principal components calculated for the geographic coordinates. Genetic isolation by distance was determined as the correlation between Euclidean distance along the averaged genetic PCs and geographic (degree) distance. Significance was assessed in a Mantel test. Finally, a third analysis of genetic structure was carried out with the software Structure v2.3.3 [38,83] using mapped loci. This method assumes Hardy-Weinberg equilibrium for the tested population and unlinked or weakly linked loci are required for clustering analysis. Before carrying out this analysis of genetic structure, we checked that the markers used were in Hardy-Weinberg equilibrium. Then, for a given EST contig, we chose a single SNP at random, to avoid the problem of LD between loci. Based on these criteria, we used a genome-wide set of 1,180 mapped SNPs for the genetic structure analysis. We carried out three runs of Structure for each tested number of groups (*K*), from 1 to 10. The correlated allele frequency model with admixture was used, with burn-in and run-length periods of 2.5x10^5^ iterations. We used the mean likelihood L(*K*) and Evanno’s delta K criterion [84] values obtained over three runs to determine whether an optimum value of *K* could be identified, as expected when discrete populations are present in the data.

### Spatial structure of diversity on chromosomes

A SNP diversity map was superimposed on the composite linkage map. We used the FGB population to test departure from Hardy-Weinberg equilibrium and to estimate three genetic diversity parameters for each SNP: minor allele frequency (MAF), observed heterozygosity (Ho) and expected heterozygosity (H_e_, Nei’s index of genetic diversity [85]. Raw data (SNP genotypes for each individual) were formatted with GenAlEx6 [86] and analyses were conducted with the GenePop package [87,88] available online at URL: http://genepop.curtin.edu.au/. Genetic diversity parameters were finally retrieved from the output of GenePop, using a PerlScript. As these three parameters were highly correlated, we considered only H_e_.

We first analyzed the spatial structure of diversity along the LGs of the composite map by variance analysis, generating a statistic that can be used to assess the covariance (i.e. correlation) between a variable of interest (here, H_e_) and the location at which it is measured (here, the position of SNP markers on the composite linkage map). The covariance calculated is equal to half the variance of the differences in the value of a metric (Z) between all pairs of points (i and j) separated by a given distance (h). This approach is often referred to as semivariance analysis in geostatistical studies (but see [89] for the confusion between the terms variance and semivariance). If pairs of points are closely located spatially and correlated, then they will have a low variance. The underlying assumption is that the difference in diversity between any two markers is a function of the distance between these markers.

The empirical variance (${\hat{\gamma }}_{h}$) was calculated as follows:

$$\begin{array}{l} {\hat{\gamma }}_{h}=1/2\mathrm{Var}\left[Z_{s_{i}}-Z_{s_{j}}\right]=\frac{1}{2N_{h}}\sum_{N_{h}}{\left(Z_{s_{i}}-Z_{s_{j}}\right)}^{2}\\ \;\mathrm{with}\;N_{h}=\left\{i,j:s_{i}-s_{j}=h\right\}, \end{array}$$

where *s*_*i*_ and *s*_*j*_ are the map positions of two SNP markers, $Z_{s_{i}}$ and $Z_{s_{j}}$ are the values of their diversity statistics (H_e_) and *N*_*h*_ is the number of paired data (SNP markers) at a distance of *h* (1 to 10 cM) or less. We calculated variance with a robust estimator, to avoid the influence of outliers, as described in [90,91]. We first estimated the empirical variogram for each LG independently, and then by pooling all the data across LGs to estimate a pangenomic variogram. We determined whether a particular value of the variance differed significantly from a random value, by carrying out permutation tests in which the H_e_ values associated with each SNP marker were randomized with respect to chromosomal position. One thousand permuted data sets were generated and the probability of finding a value higher than the observed value for a distance class was calculated from the distribution of the permuted data. We then determined whether diversity was equally distributed between LGs (presence of LGs with lower or higher overall diversity). A simple one-way ANOVA was performed, followed by a Tukey’s HSD test for multiple comparisons of means. This test compares the difference between the H_e_ values of each pair of LGs, with appropriate adjustment for multiple testing.

### Extent of linkage disequilibrium (LD) on chromosomes

LD between pairs of loci was estimated by the squared allele frequency correlation r^2^[92], based on SNP markers located on the composite map. We used the Rogers and Huff approximation for loci with unknown phases [93]. LD was calculated for all pairwise marker combinations, both within and between chromosomes. The range of minor allele frequencies in the FGB population was similar across LGs, ranging from 0.15 to 0.5, and it was assumed that this population was unstructured, as shown in the results section.

We investigated the distribution of intra-chromosomal LD over physical and genetic distances. For the estimation of short-distance (i.e. physical) LD, SNPs from the same contig (discarded for linkage map construction) were reintroduced into the LD analysis and placed at the same map position as the marker initially selected for linkage map generation. Pairwise r^2^ values were plotted against the genetic distance between the two loci (starting at 0 cM for SNPs from the same contig). We then built a null model to test for the presence of inter-chromosomal LD, by retaining only genetically linked pairs (i.e. corresponding to two different contigs) with critical values of r^2^ > 0.1 [94].

At the intragene level, LD was estimated by the squared allele frequency correlation r^2^, based on pairs of SNP belonging to the same contig, with MAF>5%. Of the 4,911 contigs studied, 248 contained two or more SNPs and were retained for the intragene LD analysis. The extent of LD was estimated by nonlinear regression analysis on the basis of intragene r^2^ values [95]. The expected values of r^2^ between pairs of adjacent sites (E(r^2^)) were estimated with the formula:

$$E\left(r^{2}\right)=\left[\frac{10+C}{\left(2+C\right)\left(11+C\right)}\right]\;\left[1+\frac{\left(3+C\right)\left(12+12C+C^{2}\right)}{n\left(2+C\right)\left(11+C\right)}\right],$$

which is valid under drift recombination equilibrium and low mutation rate and can be adjusted for sample size [96]. In this formula, C is the population recombination parameter (ρ = 4N_e_r where *N*_*e*_ is the effective population size and *r* is the recombination rate per site and per generation) and *n* is the sample size. We carried out nonlinear regression (nls function) with R software x, replacing C with C × distance (in bp) between pairs of sites, to fit this formula to our data.

### Availability of supporting data

Supporting data are available as additional files.

## Competing interests

The authors declare that they have no competing interests.

## Authors’ contributions

EC, JE and CP constructed the composite linkage map; EC, LB and CP sampled trees from the Aquitaine breeding population and analyzed the SNP data; JBL analyzed the pan-genomic distribution of recombination and genetic diversity, EM, JV, MB and CP carried out the population genetic analysis, IL and FE performed the bioinformatic analyses, FI brought its expertise in terms of implementation of genomic selection in pine breeding. CP wrote the manuscript with input from all authors, obtained funding for the research, conceived, designed and coordinated the study. All authors read and approved the final manuscript.

## Supplementary Material

Additional file 1 Alignments of the composite linkage maps obtained with LPmerge (LPM on the left) and MergeMap (MM on the right) software. Click here for file

Additional file 2 **Comparison between LPmerge and MergeMap for composite map construction.** The first table provides the two metrics for statistical testing, *i.e.* linkage group length of the composite map and root mean square error (RMSE), whereas the second table provides the result of the test. Two intermediate composite maps (G2F_F2 and G2M_F2) were constructed before the production of the final composite map (G2F_F2-G2M_F2). Click here for file

Additional file 3 a) Pairwise kinship relationships between 192 individuals of the FGB population, showing 3 pairs of trees with identical genotypic information over the 2,600 SNPs, which were therefore considered to be mislabeled in the tree archive, b) Pairwise kinship relationships between the 186 individuals of the FGB population, i.e. excluding the three abovementioned pairs. Click here for file

Additional file 4 List of SNP markers with dbSNP accession numbers, corresponding contig ID in PineContig_v2, genetic parameters in the first-generation breeding population, and linkage group assignment on the component maps. Click here for file

Additional file 5 a) Plot of genetic PC1 and PC2 and their relationship to the two geographic components, b) biplot of PCA against geographic coordinates, c) relationship between the first genetic and geographic PC (averaged per location), d) relationship between the second genetic and geographic PC (averaged per location), e) genetic distance (along the first two genetic PCs) as a function of geographic distance. Click here for file

Additional file 6 **Clustering of the 186 G0 trees of the FGB population using the Structure software.** Distribution of Evanno’s delta K values (A) and example of barplots obtained with numbers of groups K varying from 2 to 5 (A). Click here for file

Additional file 7 **Distribution of genetic diversity (H**_**e **_**values) along the 12 linkage groups of the maritime pine composite map.** Blue: one SNP in the contig, H_e_ value for the SNP; red: two SNPs in the same contig, H_e_ value for the second SNP; Green: three SNPs in the same contig, H_e_ value for the third SNP; Purple: four SNPs in the same contig, H_e_ value for the fourth SNP. Click here for file

Additional file 8 **Plot of linkage disequilibrium, measured as the squared correlation coefficient of allele frequencies (r**^**2**^**), against genetic map distance (cM) between all marker pairs in each of the 12 linkage groups (LG) of the maritime pine genome.** r^2^ was determined with the GGT 2.0 program, from the polymorphism data for 186 unrelated trees of the Aquitaine population. The 0.1 critical level of r^2^ was determined after Robbins *et al.* (2011). *J Exp Bot*, 62:1831–1845. Click here for file

Additional file 9 **Distribution of long distance intra-chromosomal linkage disequilibrium (LD) as estimated by r**^**2**^**.** This distribution was used as a null model to test the significance of inter-chromosomal LD potentially due epistatic selection. Click here for file

Additional file 10 **List of 65 pairs of markers with MAF>20% and associated linkage disequilibrium values (r**^**2**^**).** Click here for file

Additional file 11 Estimates of effective population sizes. Click here for file

Additional file 12 **Description of the three component maps from Chancerel *et al*. (2013).** Click here for file

Additional file 13 Geographic origin of the G0 trees. Click here for file

Additional file 14 Genotyping dataset (186 trees of the FGB population × 2,600 SNPs). Click here for file

Additional file 15 Bandwidth values (cM) obtained by kernel density analysis for the composite linkage map obtained with LPmerge. Click here for file
