# Supplementary material for: Genome-wide distribution of genetic diversity and linkage disequilibrium in a mass-selected population of maritime pine
Source: BMC Genomics. 2014 Mar 1;15:171. doi: 10.1186/1471-2164-15-171 (PMC4029062; doi:10.1186/1471-2164-15-171)
Supplement: Additional file 1 — Alignments of the composite linkage maps obtained with LPmerge (LPM on the left) and MergeMap (MM on the right) software. [file 1471-2164-15-171-S1.PDF]

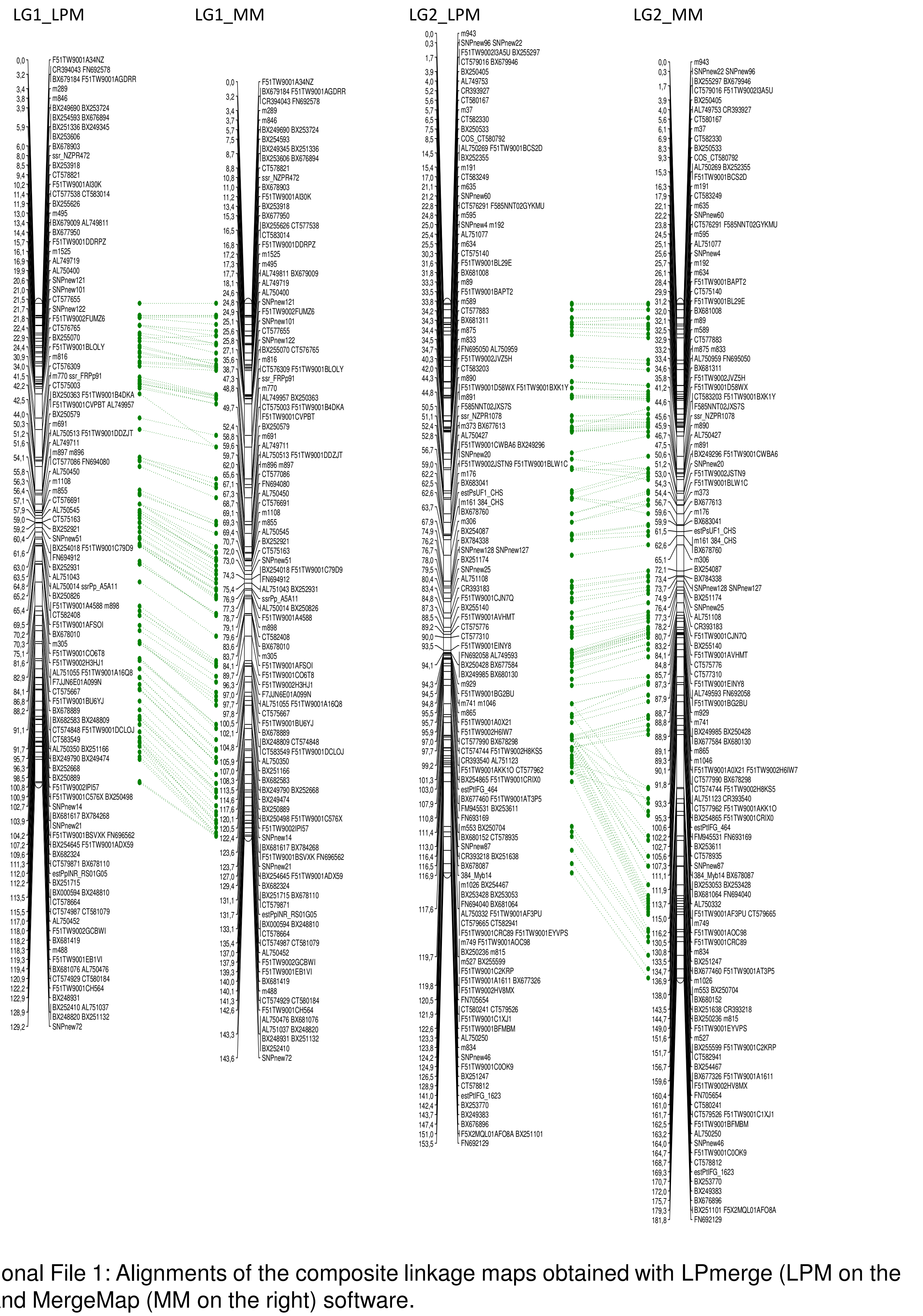

Additional File 1: Alignments of the composite linkage maps obtained with LPmerge (LPM on the left) and MergeMap (MM on the right) software.

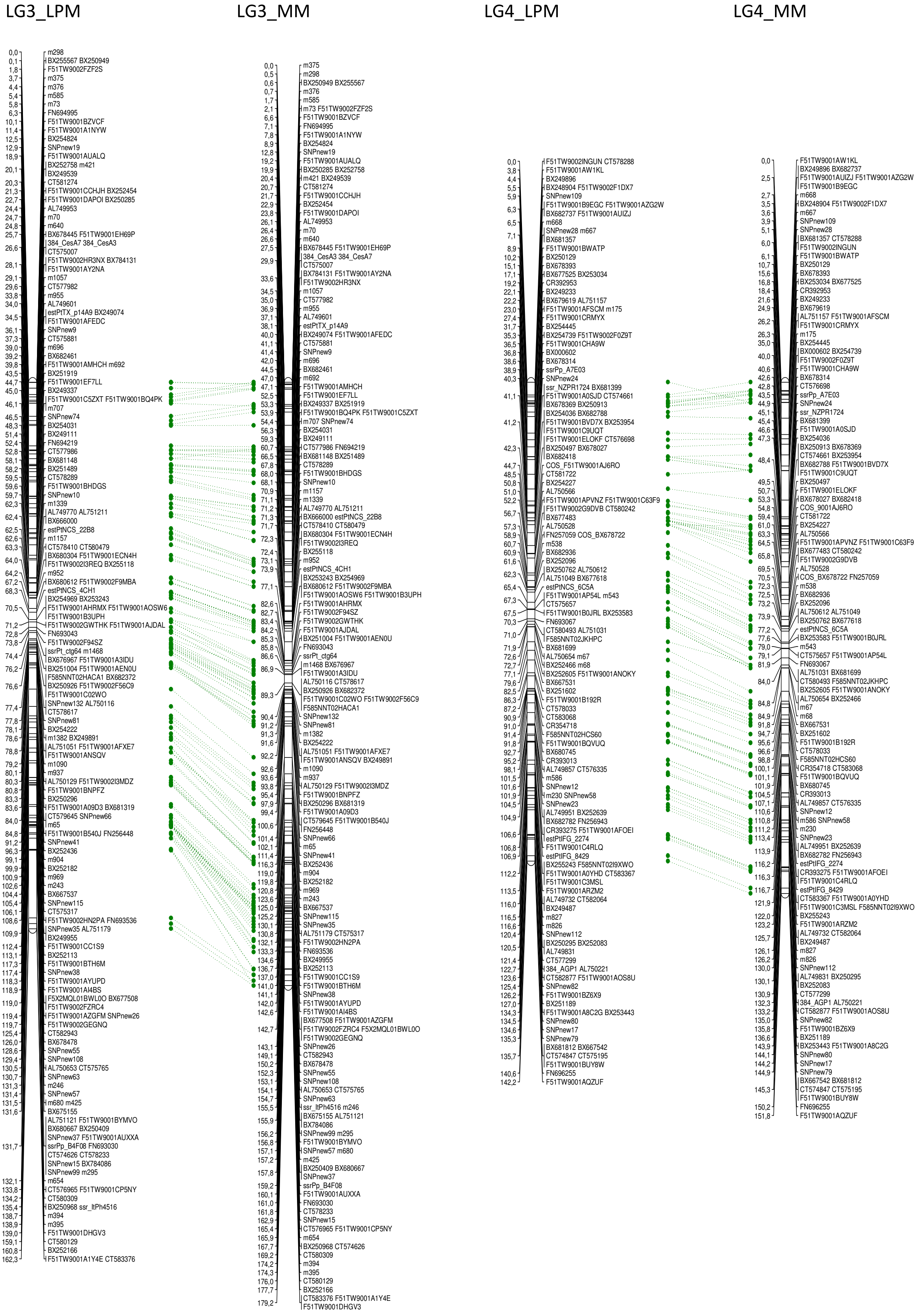

LG3_LPM
LG3_MM
LG4_LPM
LG4_MM

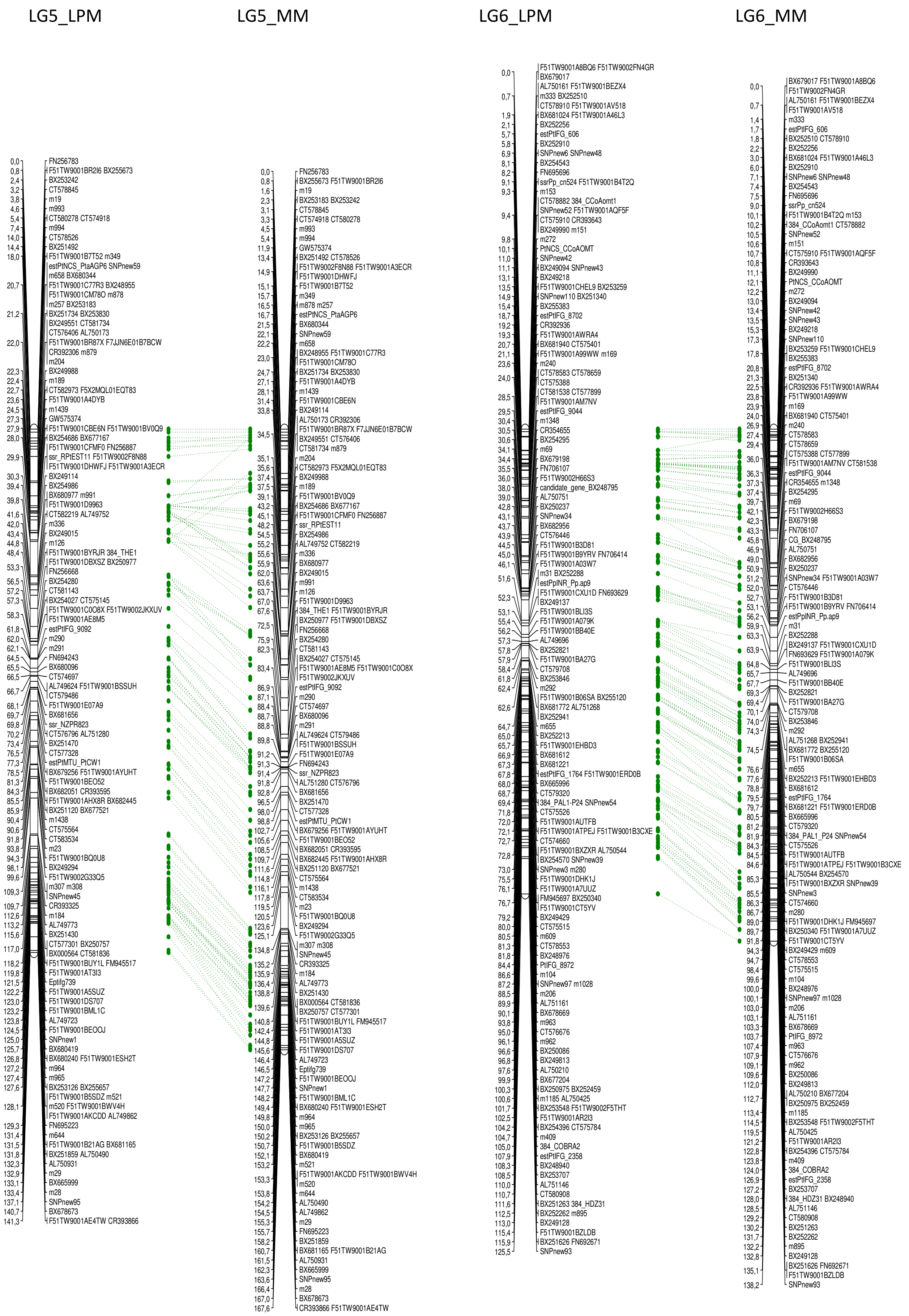
LG5_LPM
LG5_MM
LG6_LPM
LG6_MM

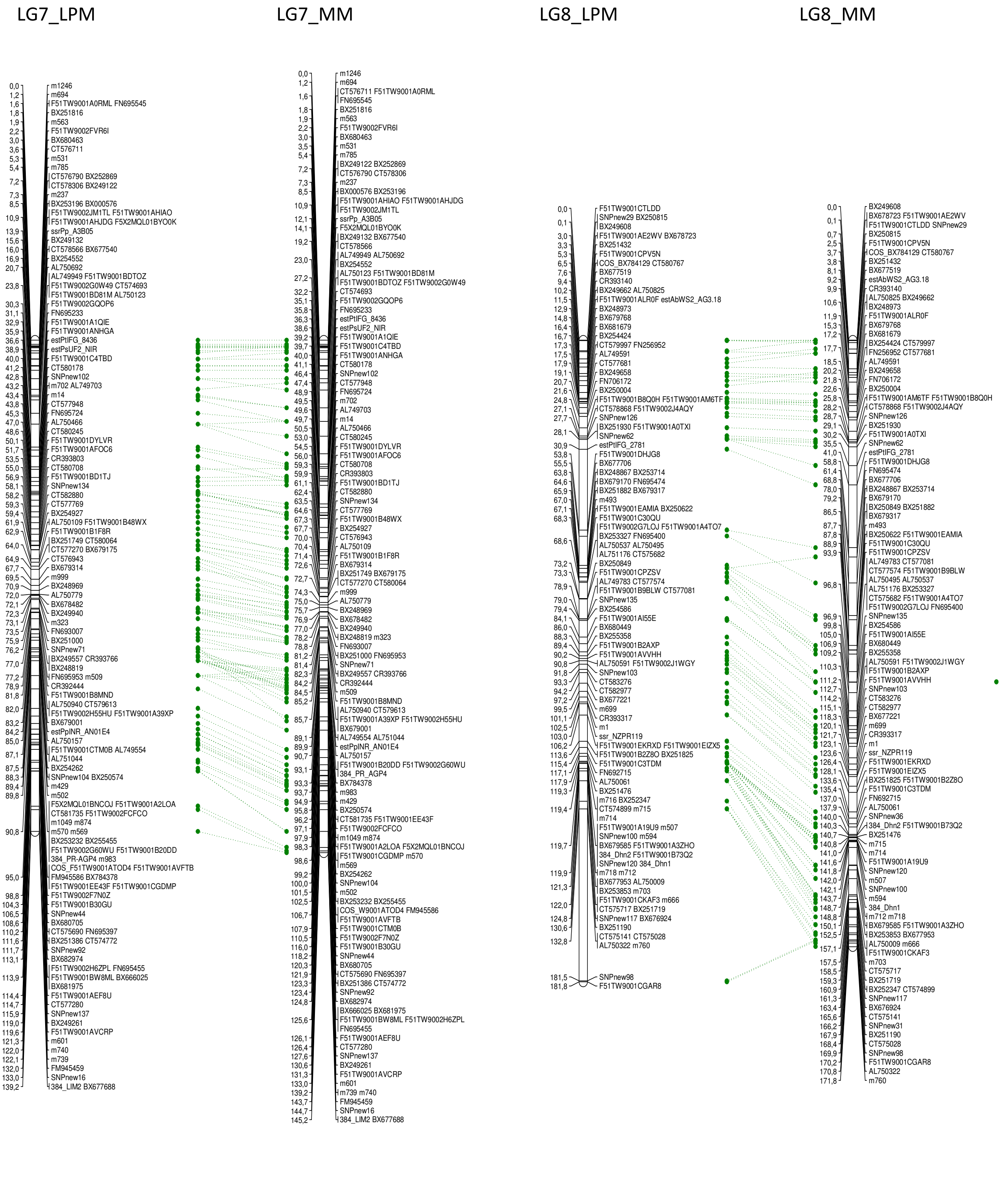

LG7_LPM
LG7_MM
LG8_LPM
LG8_MM

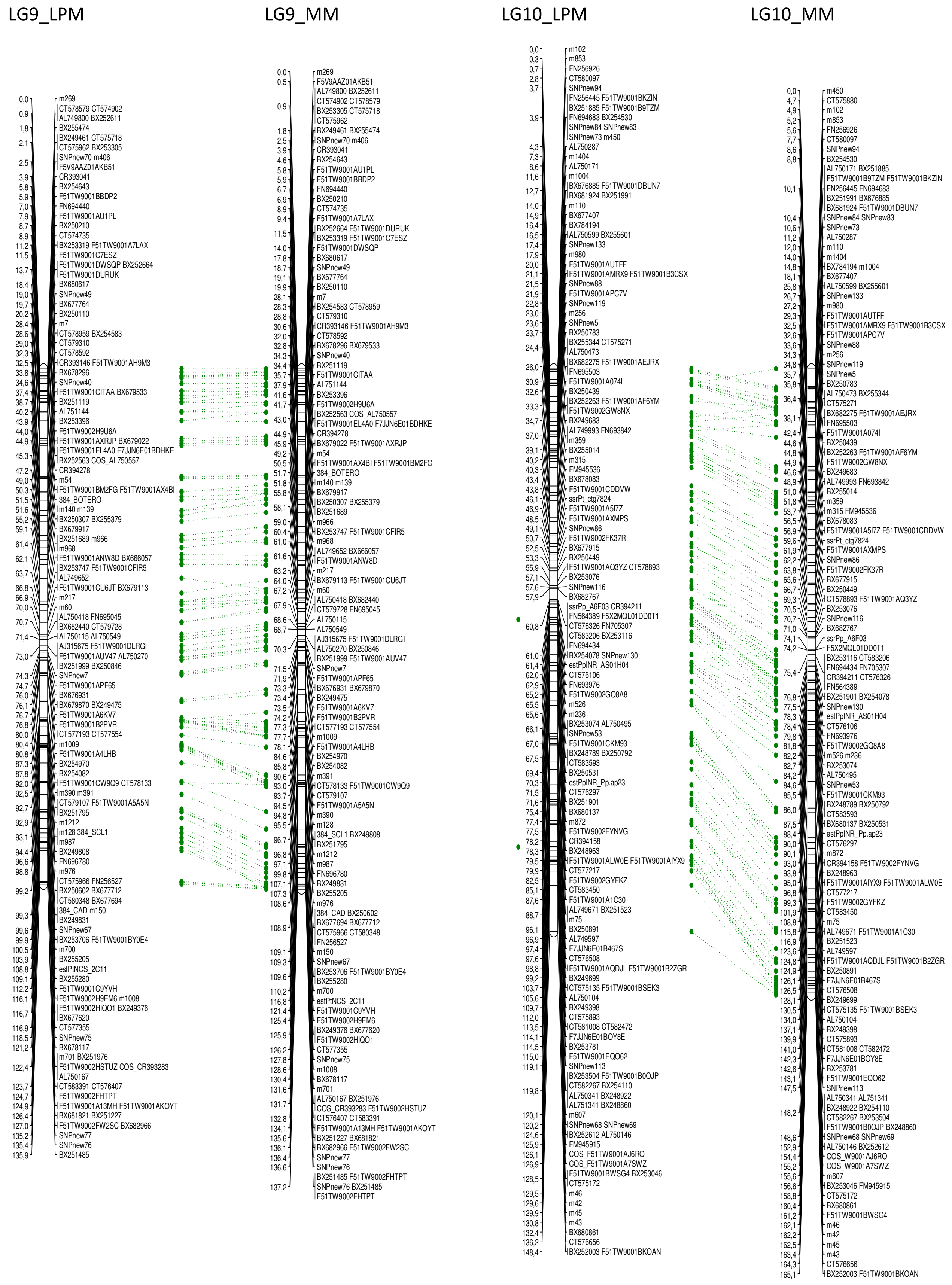
LG9_LPM
LG9_MM
LG10_LPM
LG10_MM

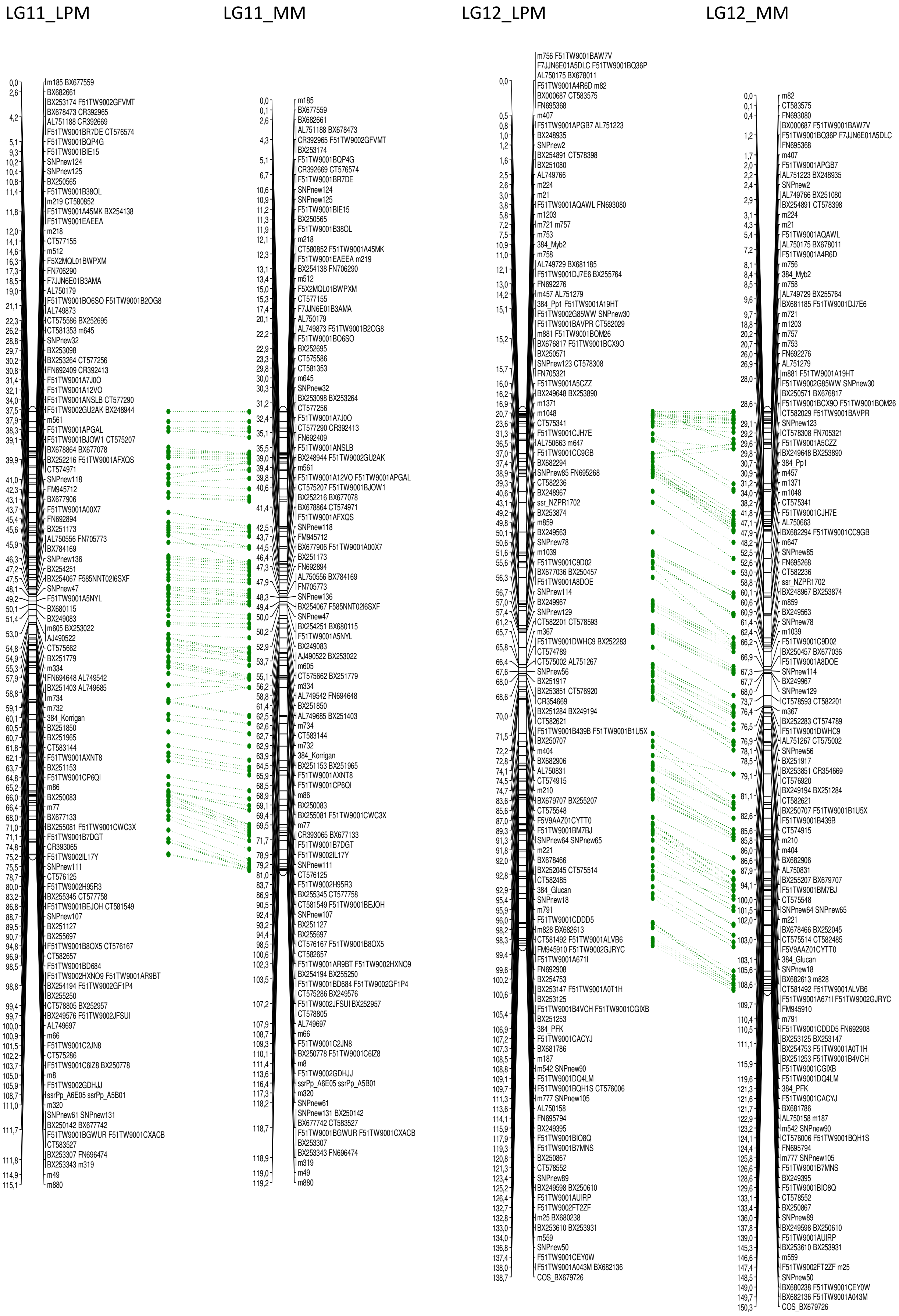
LG11_LPM
LG11_MM
LG12_LPM
LG12_MM
