# Supplementary material for: Genome-wide distribution of genetic diversity and linkage disequilibrium in a mass-selected population of maritime pine
Source: BMC Genomics. 2014 Mar 1;15:171. doi: 10.1186/1471-2164-15-171 (PMC4029062; doi:10.1186/1471-2164-15-171)
Supplement: Additional file 2 — Comparison between LPmerge and MergeMap for composite map construction. The first table provides the two metrics for statistical testing, i.e. linkage group length of the composite map and root mean square error (RMSE), whereas the second table provides the result of the test. Two intermediate composite maps (G2F_F2 and G2M_F2) were constructed before the production of the final composite map (G2F_F2-G2M_F2). [file 1471-2164-15-171-S2.DOCX]

**Additional File 2:** Comparison between LPmerge and MergeMap for composite map construction. The first table provides the two metrics for statistical testing, *i.e.* linkage group length of the composite map and root mean square error (RMSE), whereas the second table provides the result of the test. Two intermediate composite maps (G2F_F2 and G2M_F2) were constructed before the production of the final composite map (G2F_F2-G2M_F2).

|  | **Composite G2F_ F2** | | | | | | | | **Composite G2M_F2** | | | | | | | | **Composite G2F_F2- G2M_F2** | | | | | | | |
| --- | --- | --- | --- | --- | --- | --- | --- | --- | --- | --- | --- | --- | --- | --- | --- | --- | --- | --- | --- | --- | --- | --- | --- | --- |
|  |  |  | **LPmerge** | | | | **MergeMap** | |  | | **LPmerge** | | | | **MergeMap** | |  | | **LPmerge** | | | | **MergeMap** | |
|  | **Nb of common markers in individual maps** | **Nb of markers in the composite map** | **Lenght of the composite map** | **Nb of neighbor used (k)** | **Conflict order detected (Nb)** | **RMSE F2 / RMSE G2F** | **Lenght of the composite map** | **RMSE F2 / RMSE G2F** | **Nb of common markers in individual maps** | **Nb of markers in the composite map** | **Lenght of the composite map** | **Nb of neighbor used (k)** | **Conflict order detected (Nb)** | **RMSE F2 / RMSE G2M** | **Lenght of the composite map** | **RMSE F2 / RMSE G2F** | **Nb of common markers in intermediate composite maps *** | **Nb of markers in the composite map** | **Lenght of the composite map** | **Nb of neighbor used (k)** | **Conflict order detected (Nb)** | **RMSE G2F_F2 / RMSE G2M_F2** | **Lenght of the composite map** | **RMSE G2F_F2 / RMSE G2M_F2** |
|  |  |  |  |  |  |  |  |  |  |  |  |  |  |  |  |  |  |  |  |  |  |  |  |  |
| LG1 | 13 | 104 | 129 | 3 | No | 2.5 / 1.4 | 136 | 8.1 / 5.1 | 16 | 100 | 126 | 3 | No | 4.3 / 1.4 | 133 | 4.3 / 9.7 | 68 | 134 | 129 | 4 | Yes (1) | 3.6 / 3.6 | 143.5 | 4.3 / 8.2 |
| LG2 | 24 | 126 | 122 | 1 | Yes (5) | 7 / 14.5 | 168 | 13.8 / 16.1 | 19 | 120 | 153.5 | 6 | Yes (2) | 3 / 2.1 | 153 | 1.8 / 5.5 | 90 | 156 | 153.5 | 8 | Yes (2) | 6 / 5.7 | 182 | 3.8 / 13 |
| LG3 | 15 | 157 | 163 | 3 | No | 12.7 / 5.7 | 178 | 7 / 24.8 | 26 | 154 | 142 | 3 | Yes (3) | 10.2 / 4 | 165 | 1.6 / 15 | 125 | 186 | 162 | 8 | Yes (4) | 3.9 / 3.6 | 179 | 1.8 / 6.6 |
| LG4 | 13 | 121 | 138 | 5 | Yes (2) | 1.5 / 1.85 | 140 | 2.2 / 3.7 | 20 | 127 | 142 | 5 | Yes (2) | 4.5 / 4.1 | 152 | 12.3 / 7.5 | 100 | 148 | 142 | 1 | Yes (3) | 3.4 / 2.8 | 152 | 9.4 / 2 |
| LG5 | 11 | 119 | 156 | 4 | Yes (1) | 2 / 3 | 156 | 1 / 16.1 | 19 | 133 | 129 | 1 | Yes (2) | 22 / 7.1 | 164 | 6.6 / 18.3 | 97 | 155 | 141 | 6 | Yes (3) | 10.5 / 11 | 168 | 7.5 / 3.85 |
| LG6 | 14 | 134 | 125 | 3 | Yes (1) | 5.6 / 5.8 | 129 | 2.65 / 11.4 | 19 | 128 | 125.5 | 2 | Yes (2) | 4.1 / 4 | 133 | 2.2 / 6.5 | 98 | 164 | 125.5 | 8 | Yes (4) | 2.1 / 3 | 138 | 4 / 3.3 |
| LG7 | 12 | 122 | 142 | 3 | Yes (1) | 4.3 / 3.4 | 141 | 1.1 / 7.3 | 20 | 121 | 127 | 1 | Yes (4) | 9 / 9.7 | 147 | 5.6 / 18.5 | 97 | 146 | 139 | 7 | Yes (2) | 3.8 / 4 | 145 | 4.3 / 1.8 |
| LG8 | 17 | 92 | 182 | 3 | No | 37.3 / 13.5 | 172 | 9.8 / 43.5 | 13 | 98 | 182 | 4 | Yes (2) | 11.7 / 29.3 | 174 | 7.5 / 34.9 | 68 | 121 | 182 | 4 | Yes (2) | 10.7 / 19.4 | 172 | 0.9 / 3.15 |
| LG9 | 15 | 129 | 130 | 3 | Yes (1) | 5.6 / 9.6 | 141 | 2.9 / 14.3 | 24 | 123 | 136 | 4 | Yes (3) | 9.7 / 10.9 | 138 | 3.3 / 19.5 | 98 | 154 | 136 | 5 | Yes (2) | 3.4 / 4.9 | 137 | 2.3 / 2.5 |
| LG10 | 17 | 115 | 148 | 2 | No | 10.5/ 2.3 | 154 | 3.5 / 13.4 | 18 | 123 | 123 | 2 | Yes (1) | 8.4 / 2.9 | 161 | 8.7 / 19.5 | 89 | 158 | 148 | 7 | Yes (1) | 3.4 / 3.9 | 165 | 7.1 / 2.6 |
| LG11 | 27 | 115 | 115 | 4 | Yes (2) | 5.4 / 6 | 120 | 3.3 / 9.4 | 26 | 129 | 115 | 3 | Yes (2) | 1.5 / 4.5 | 116 | 0.6 / 6.6 | 95 | 149 | 115 | 7 | Yes (1) | 2 / 2.3 | 119 | 1.3 / 2.3 |
| LG12 | 20 | 138 | 144 | 5 | Yes (6) | 5.7 / 6 | 148 | 10.7 / 5.6 | 20 | 138 | 139 | 3 | No | 3.5 / 3.7 | 146 | 4.35 / 9 | 109 | 167 | 139 | 8 | Yes (2) | 8.6 / 1.8 | 150 | 4.8 / 4.8 |
| **TOTAL** | **198** | **1472** | **1694** |  |  |  | **1782** |  | **240** | **1494** | **1640** |  |  |  | **1781** |  | **1134** | **1838** | **1712** |  |  |  | **1850.5** |  |

* As the F2 map was taken twice for intermediate composite map construction, the numbers of common markers between intermediate composite maps does not reflect the real number of common markers between G2 and F2 maps.

Wilcoxon rank tests for comparison between LPmerge and MergeMap. For each hypothesis tested, P-values are indicated and * show significant results at a type I error risk of 5%.

|  | Intermediate composite maps | | Final composite map |
| --- | --- | --- | --- |
| Tested hypothesis | G2F_F2 | G2M_F2 | G2F_F2-G2M_F2 |
| i/ Difference in map length | P = 0.035* | P = 0.016* | P = 0.007* |
|  | > for MergeMap | > for MergeMap | > for MergeMap |
| ii/ Difference in RMSEs | P = 0.008* | P = 0.0063* | P = 0.23 |
|  | > for MergeMap | > for MergeMap |  |
| iii/ RMSE with LPmerge | P=0.71 | P=0.6 | P=0.38 |
|  | G2F = F2 | G2M = F2 |  |
| iii/ RMSE with MergeMap | P=0.01* | P=0.006* | P=0.87 |
|  | > G2F | > G2M |  |
