# Supplementary material for: Genome-wide distribution of genetic diversity and linkage disequilibrium in a mass-selected population of maritime pine
Source: BMC Genomics. 2014 Mar 1;15:171. doi: 10.1186/1471-2164-15-171 (PMC4029062; doi:10.1186/1471-2164-15-171)

**Additional File 3**: a) Pairwise kinship relationships between 192 individuals of the FGB population, showing 3 pairs of trees with identical genotypic information over the 2,600 SNPs, which were therefore considered to be mislabeled in the tree archive, b) Pairwise kinship relationships between the 186 individuals of the FGB population, i.e. excluding the three abovementioned pairs.

a/


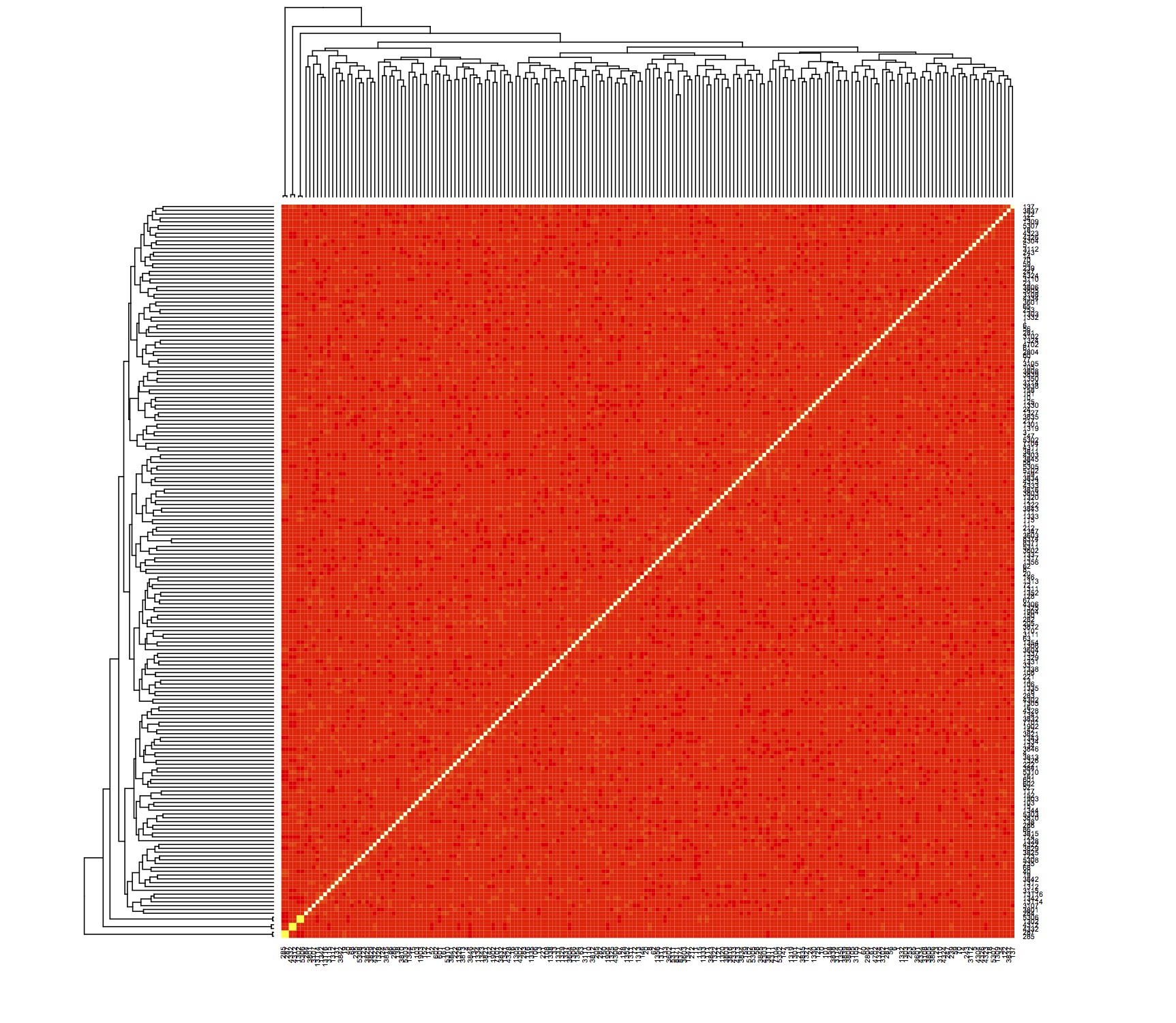


b/


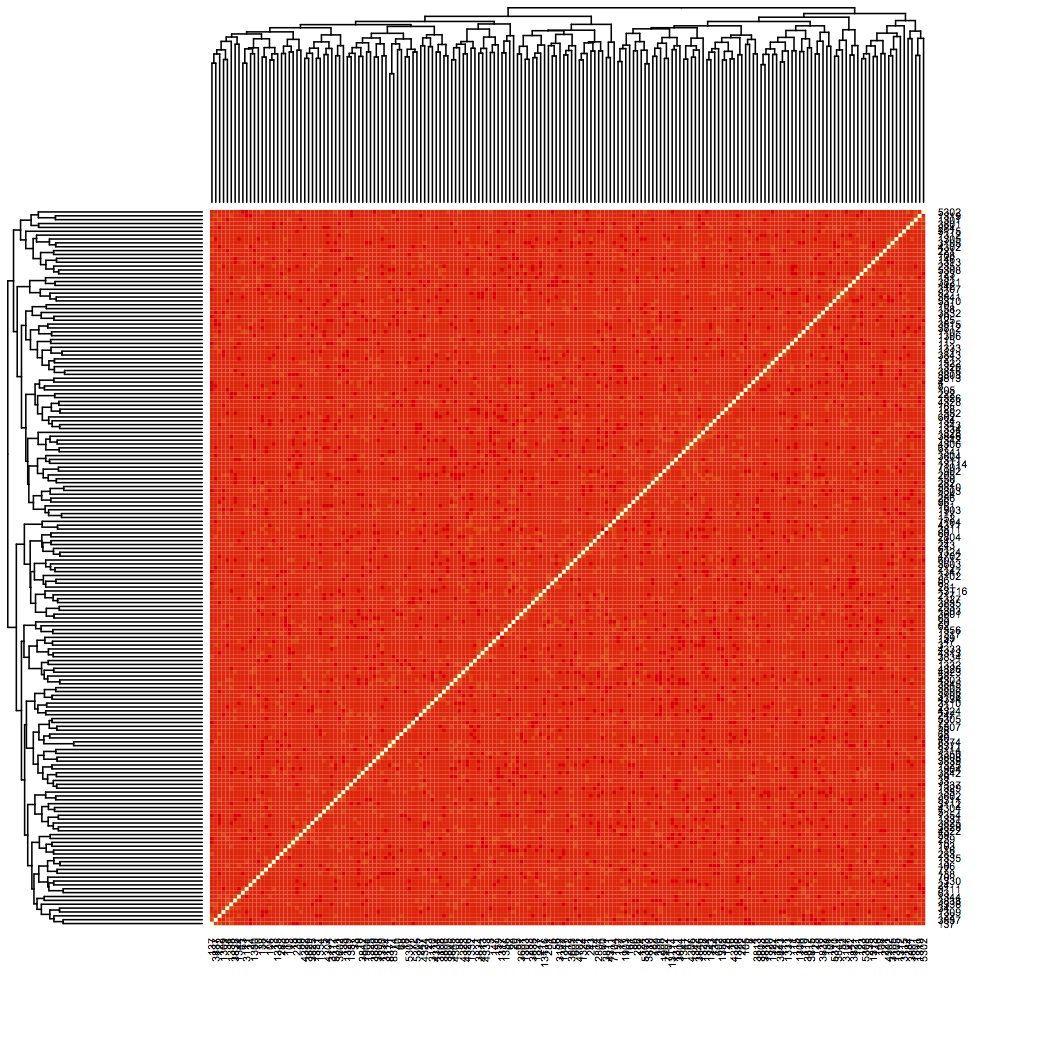

Supplement: Additional file 3 — a) Pairwise kinship relationships between 192 individuals of the FGB population, showing 3 pairs of trees with identical genotypic information over the 2,600 SNPs, which were therefore considered to be mislabeled in the tree archive, b) Pairwise kinship relationships between the 186 individuals of the FGB population, i.e. excluding the three abovementioned pairs. [file 1471-2164-15-171-S3.DOC]
