# Supplementary material for: Genome-wide distribution of genetic diversity and linkage disequilibrium in a mass-selected population of maritime pine
Source: BMC Genomics. 2014 Mar 1;15:171. doi: 10.1186/1471-2164-15-171 (PMC4029062; doi:10.1186/1471-2164-15-171)
Supplement: Additional file 5 — a) Plot of genetic PC1 and PC2 and their relationship to the two geographic components, b) biplot of PCA against geographic coordinates, c) relationship between the first genetic and geographic PC (averaged per location), d) relationship between the second genetic and geographic PC (averaged per location), e) genetic distance (along the first two genetic PCs) as a function of geographic distance. [file 1471-2164-15-171-S5.DOC]

**Additional File 5**: a) Plot of genetic PC1 and PC2 and their relationship to the two geographic components, b) biplot of PCA against geographic coordinates, c) relationship between the first genetic and geographic PC (averaged per location), d) relationship between the second genetic and geographic PC (averaged per location), e) genetic distance (along the first two genetic PCs) as a function of geographic distance.

a/

b/

c/

d/

e/
