# Supplementary material for: Genome-wide distribution of genetic diversity and linkage disequilibrium in a mass-selected population of maritime pine
Source: BMC Genomics. 2014 Mar 1;15:171. doi: 10.1186/1471-2164-15-171 (PMC4029062; doi:10.1186/1471-2164-15-171)

**Additional File 6:** Clustering of the 186 G0 trees of the FGB population using the Structure software. Distribution of Evanno’s delta K values (A) and example of barplots obtained with numbers of groups K varying from 2 to 5 (A).

A

| K | Moy LK | Moy VarK | LK-L(K-1) | LK/VarK |
| --- | --- | --- | --- | --- |
| 1 | -258619.30 | 583.90 | NA | -442.92 |
| 2 | -258125.77 | 1623.93 | 493.53 | -158.95 |
| 3 | -257758.73 | 3301.70 | 367.03 | -78.07 |
| 4 | -257451.23 | 5382.63 | 307.50 | -47.83 |
| 5 | -257159.53 | 6845.23 | 291.70 | -37.57 |
| 6 | -257049.53 | 9122.67 | 110.00 | -28.18 |
| 7 | -256914.30 | 22980.77 | 135.23 | -11.18 |
| 8 | -256691.27 | 12648.33 | 223.03 | -20.29 |
| 9 | -256678.07 | 13255.70 | 13.20 | -19.36 |
| 10 | -256621.05 | 22338.60 | 57.02 | -11.49 |


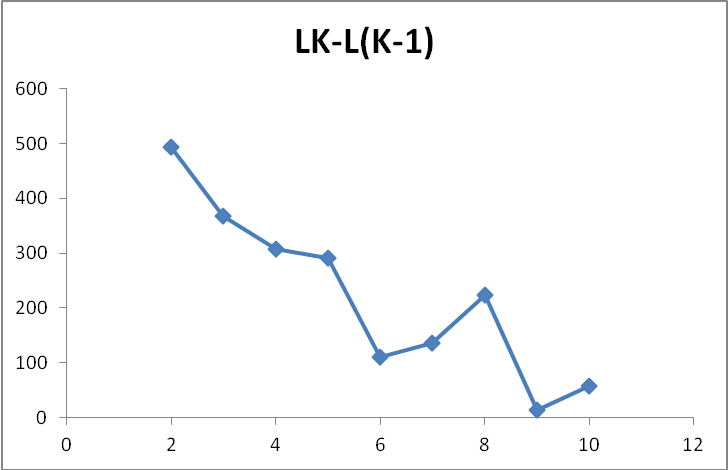


B


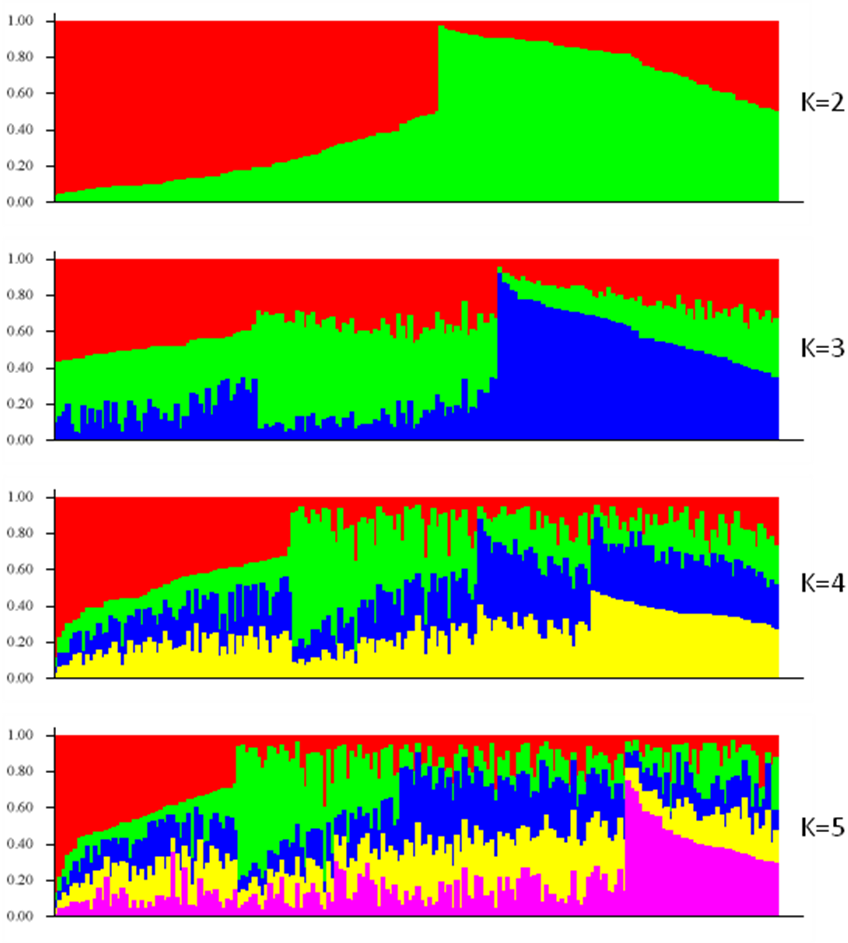

Supplement: Additional file 6 — Clustering of the 186 G0 trees of the FGB population using the Structure software. Distribution of Evanno’s delta K values (A) and example of barplots obtained with numbers of groups K varying from 2 to 5 (A). [file 1471-2164-15-171-S6.DOC]
