# Supplementary figures and images for: Genome-wide distribution of genetic diversity and linkage disequilibrium in a mass-selected population of maritime pine
Source: BMC Genomics. 2014 Mar 1;15:171. doi: 10.1186/1471-2164-15-171 (PMC4029062; doi:10.1186/1471-2164-15-171)

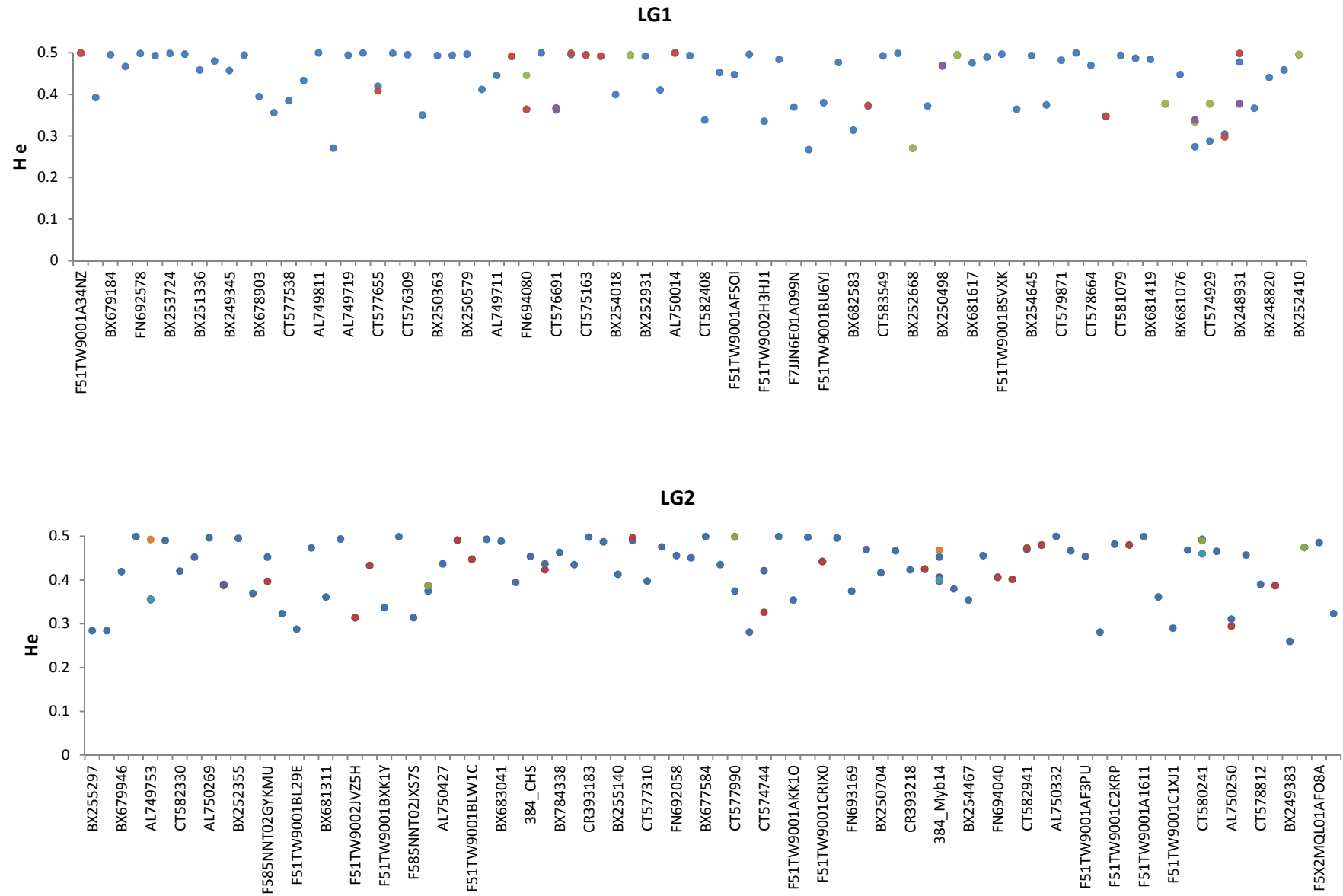

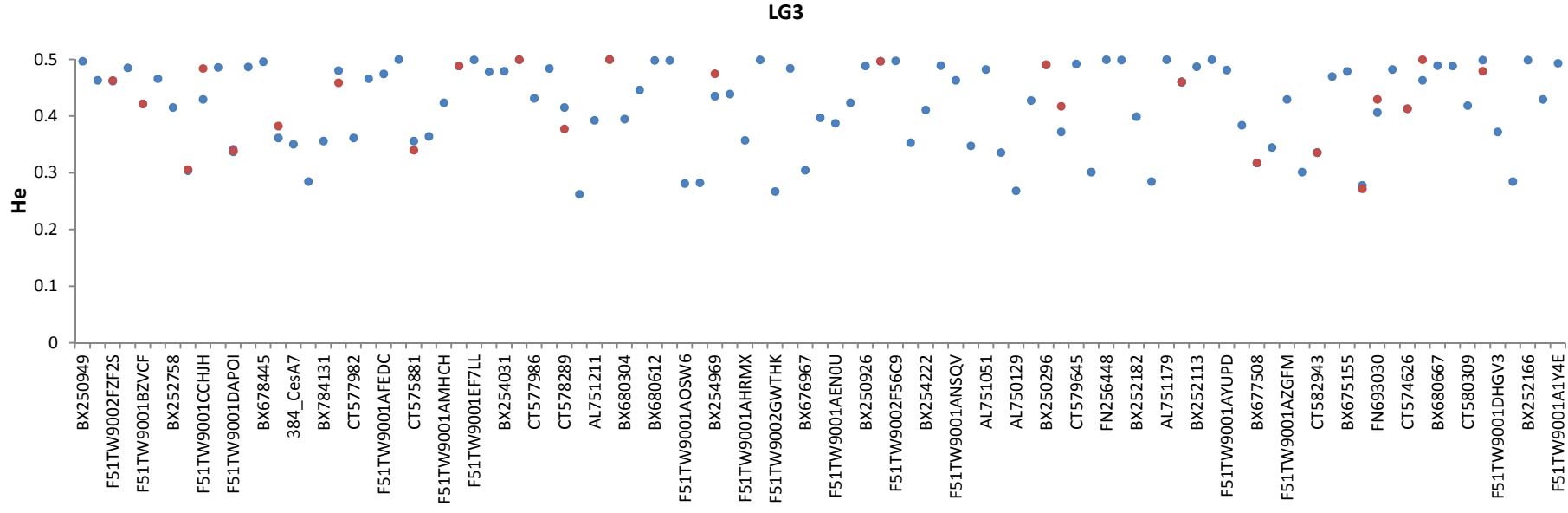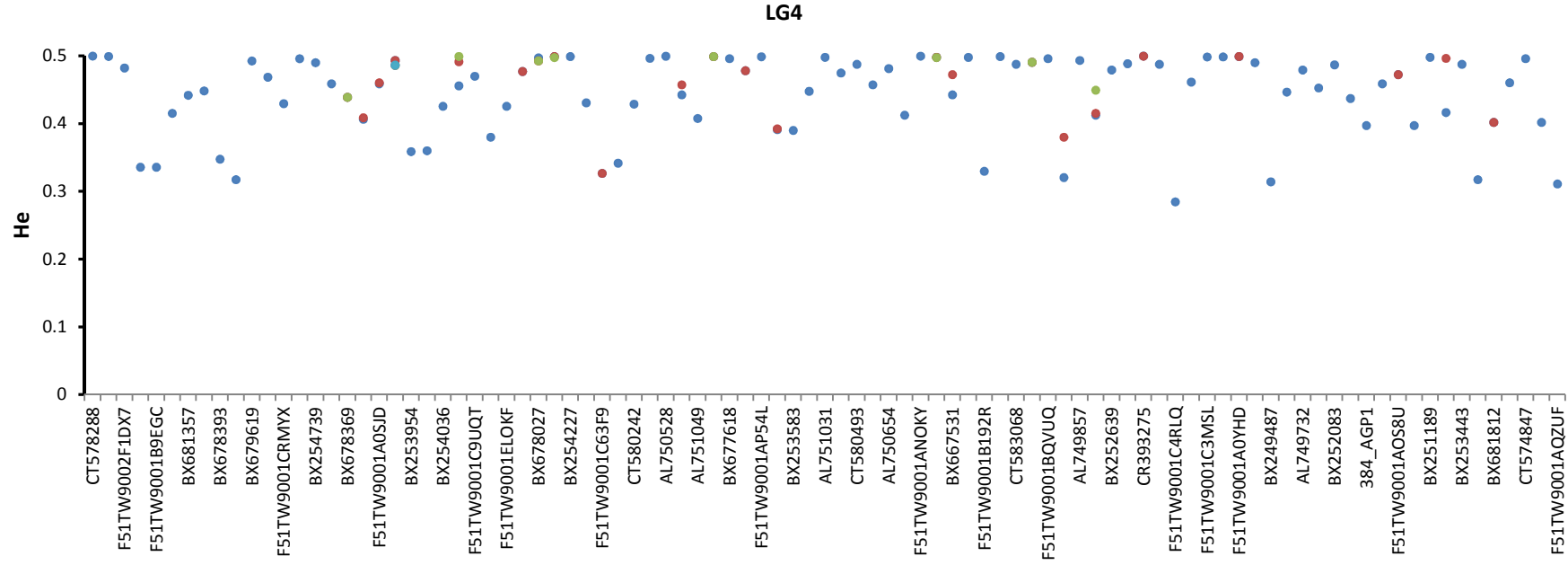

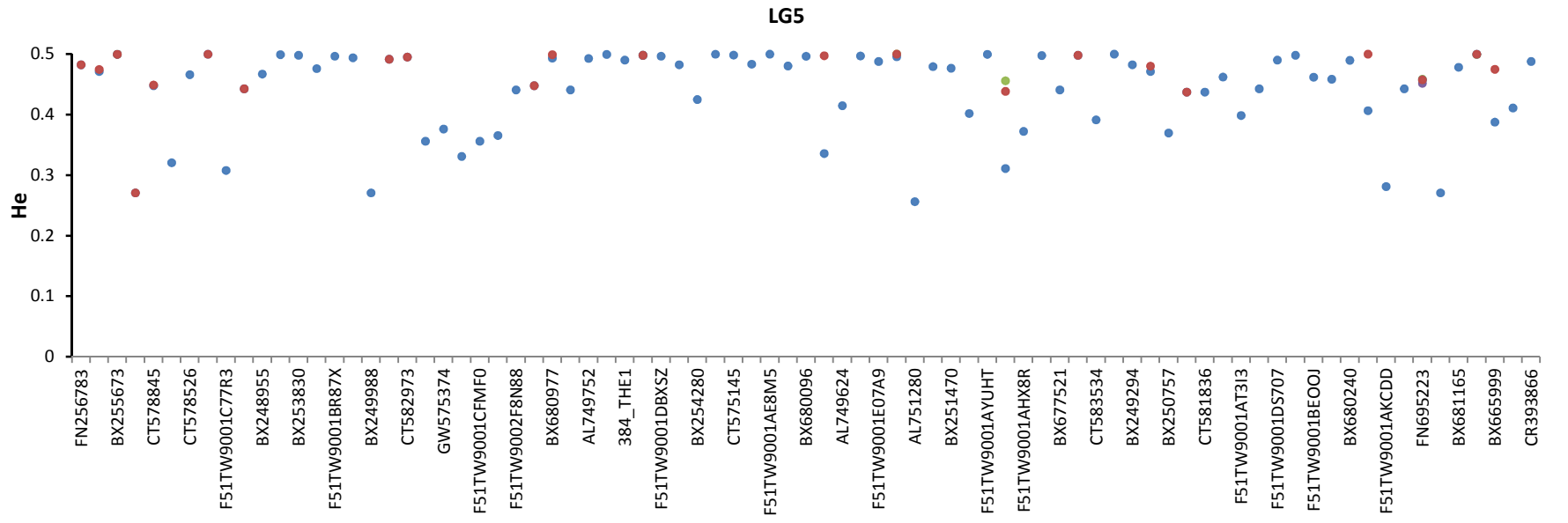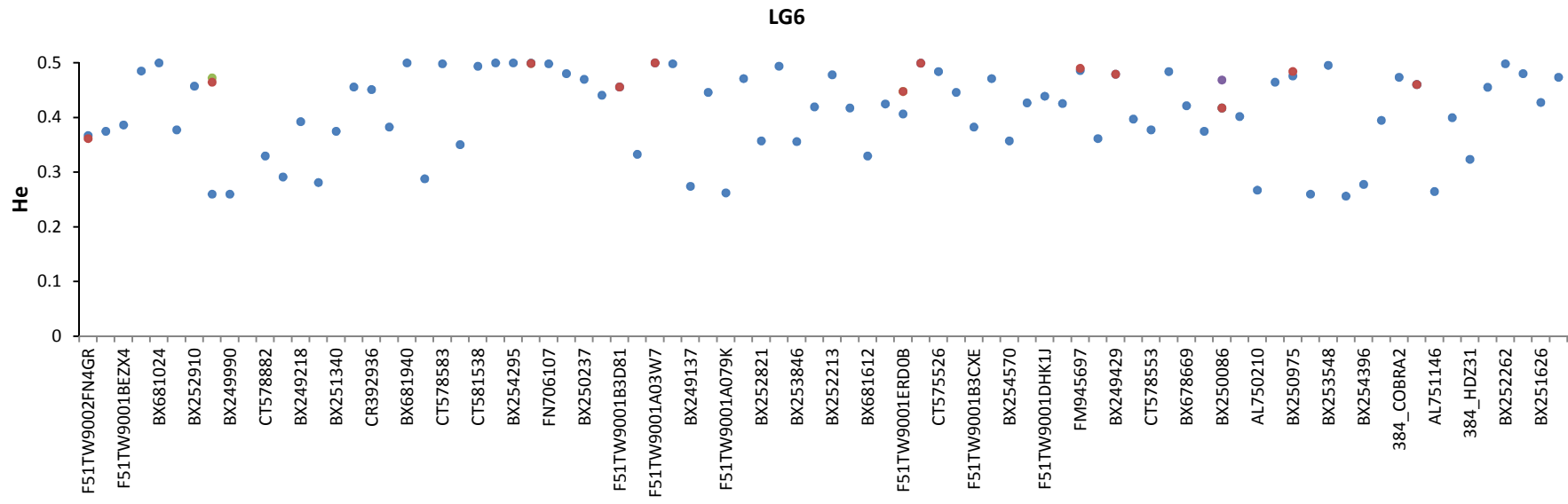

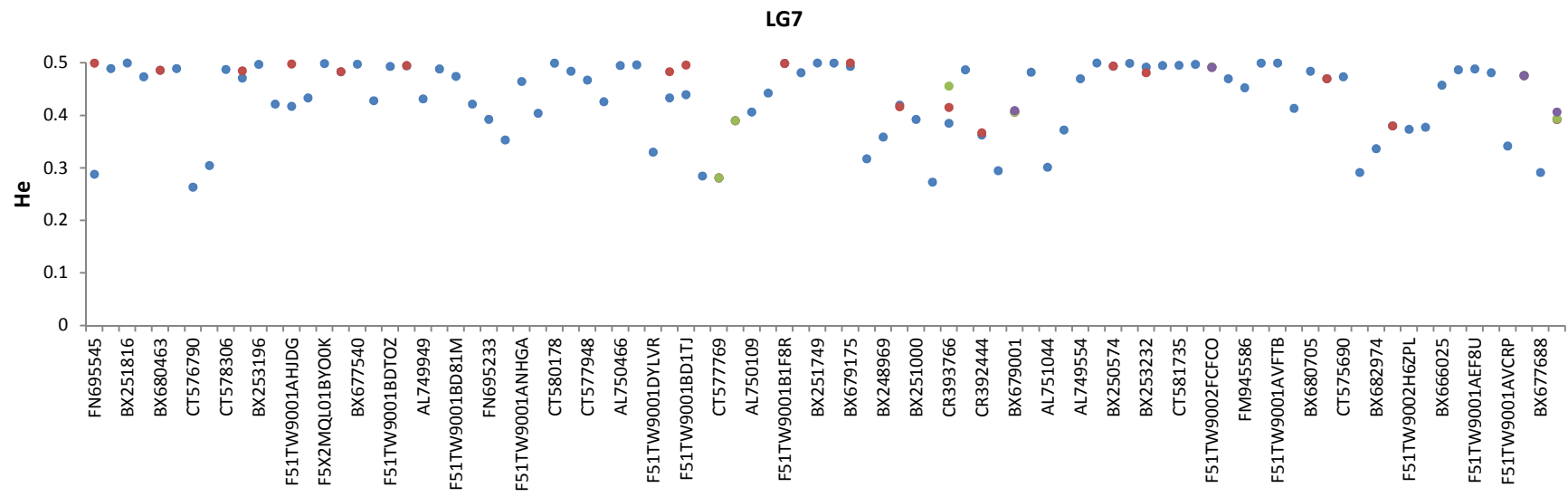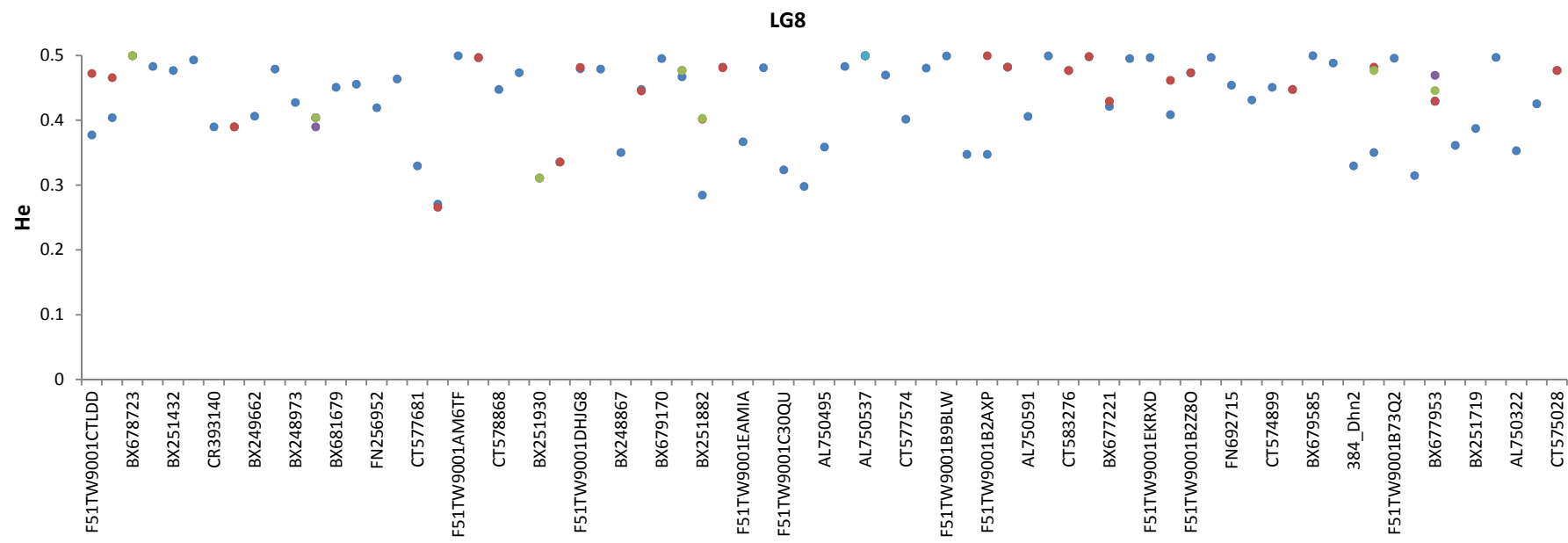

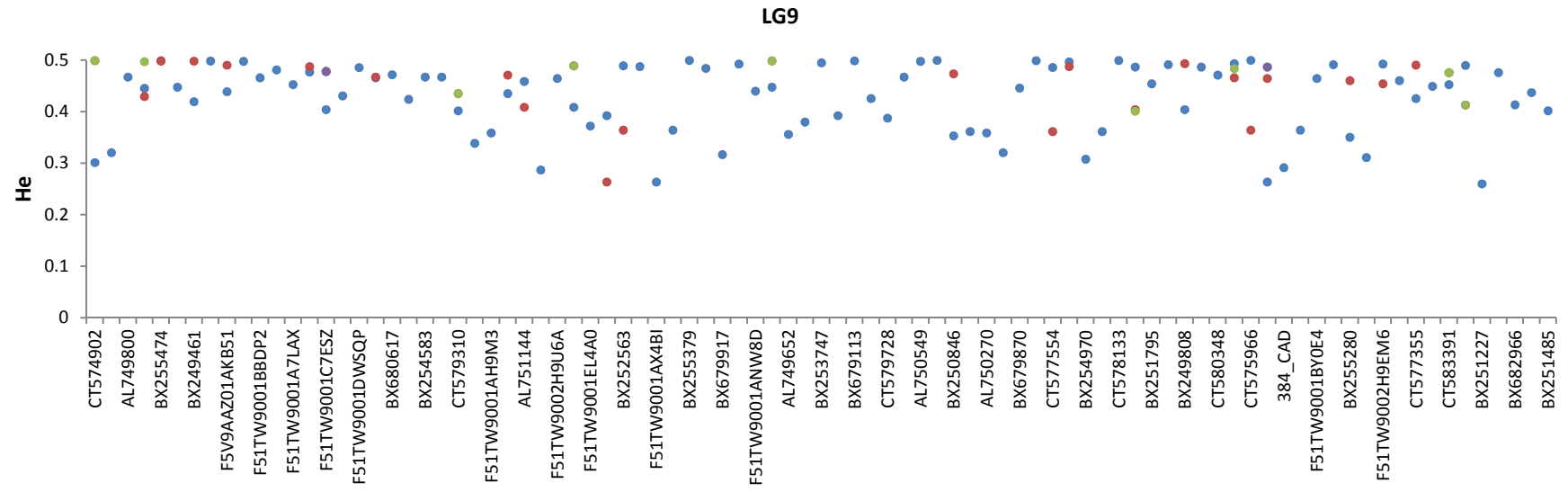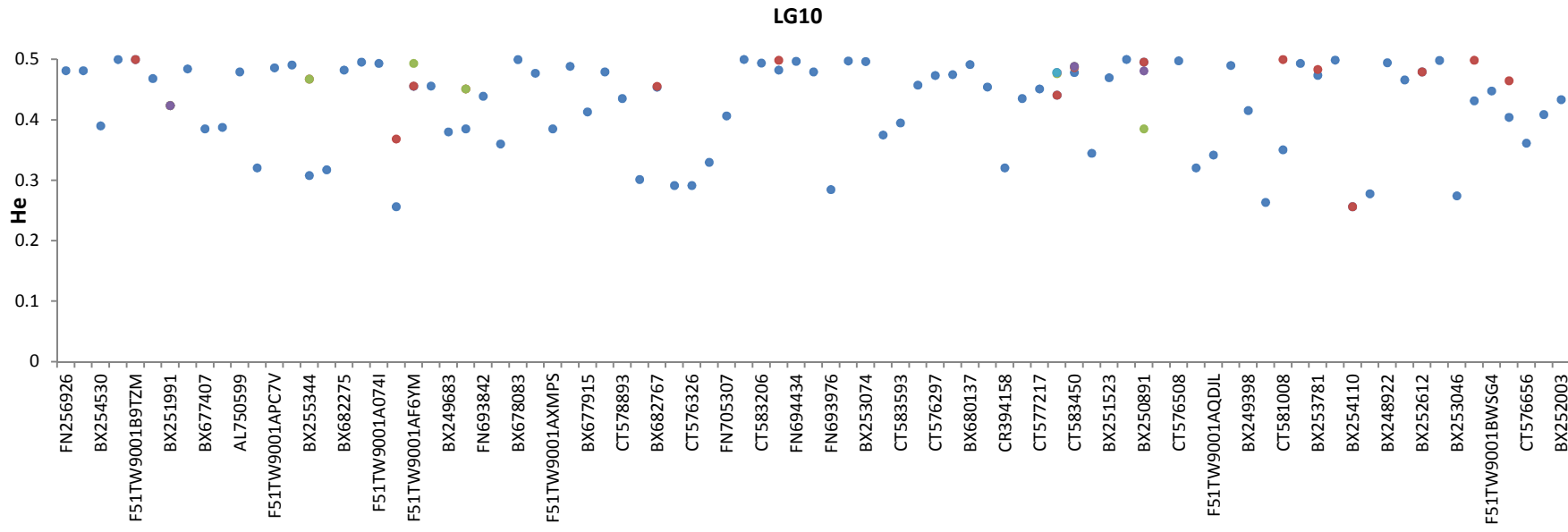

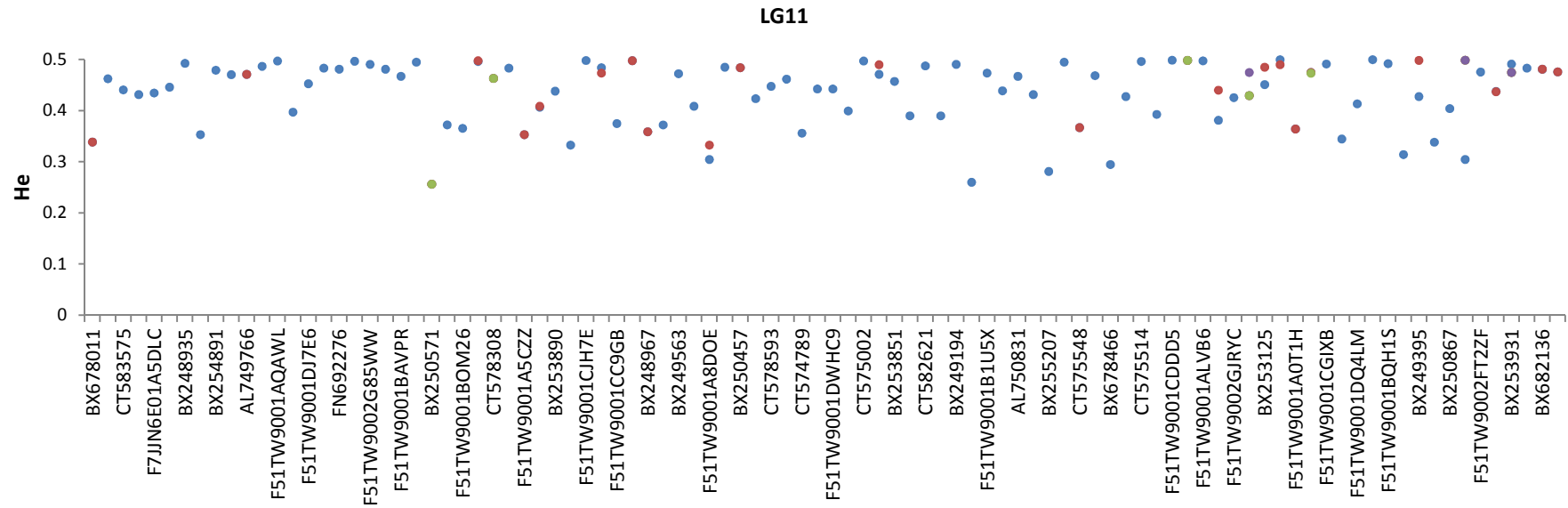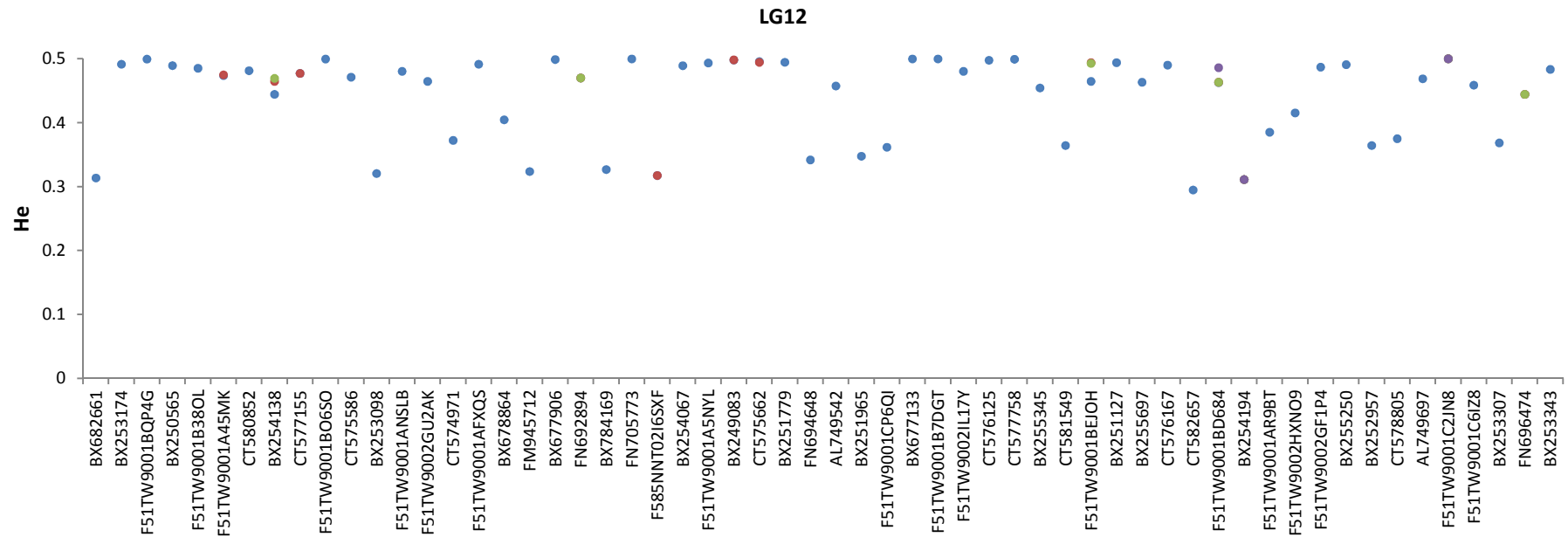

Supplement: Additional file 7 — Distribution of genetic diversity (H e values) along the 12 linkage groups of the maritime pine composite map. Blue: one SNP in the contig, He value for the SNP; red: two SNPs in the same contig, He value for the second SNP; Green: three SNPs in the same contig, He value for the third SNP; Purple: four SNPs in the same contig, He value for the fourth SNP. [file 1471-2164-15-171-S7.PDF]
