# Supplementary material for: Genome-wide distribution of genetic diversity and linkage disequilibrium in a mass-selected population of maritime pine
Source: BMC Genomics. 2014 Mar 1;15:171. doi: 10.1186/1471-2164-15-171 (PMC4029062; doi:10.1186/1471-2164-15-171)

**Additional File 8**: **Plot of linkage disequilibrium, measured as the squared correlation coefficient of allele frequencies** (r^2^), against genetic map distance (cM) between all marker pairs in each of the 12 linkage groups (LG) of the maritime pine genome. r^2^ was determined with the GGT 2.0 program, from the polymorphism data for 186 unrelated trees of the Aquitaine population. The 0.1 critical level of r^2^ was determined after Robbins *et al.* (2011). *J Exp Bot*, **62**:1831-1845.

LG1

LG2

LG3


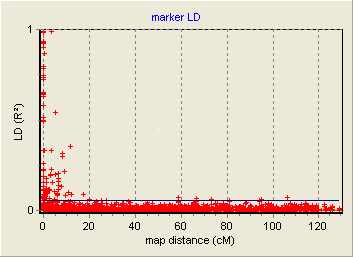

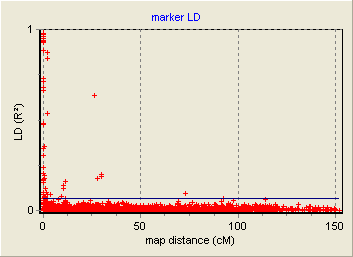

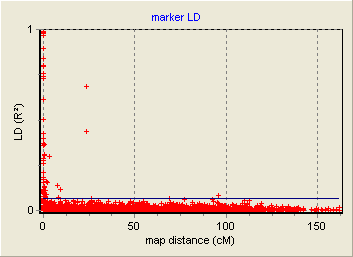


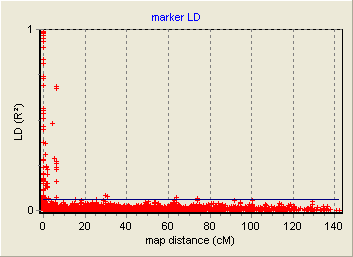

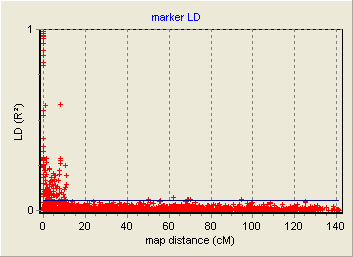

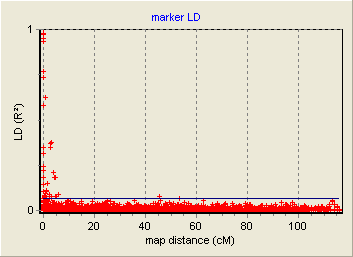


LG4

LG5

LG6

LG7

LG8

LG9


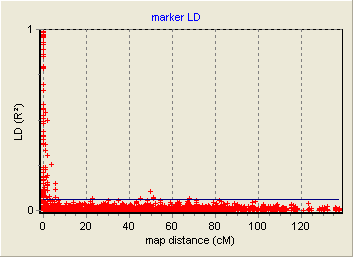

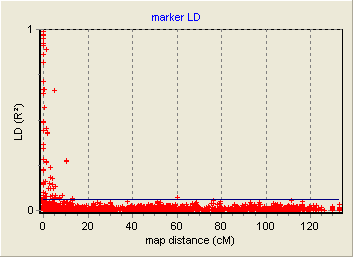

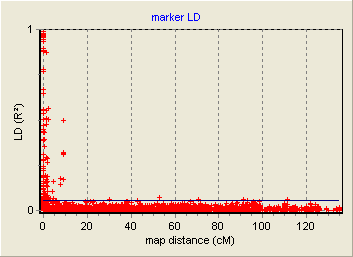


LG10

LG11

LG12


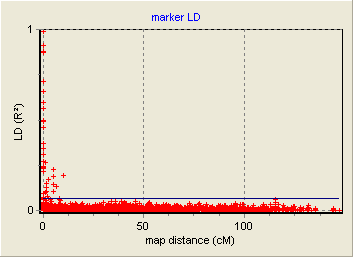

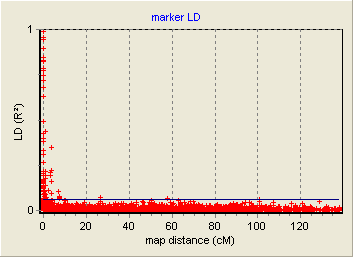

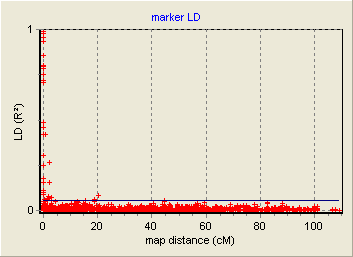

Supplement: Additional file 8 — Plot of linkage disequilibrium, measured as the squared correlation coefficient of allele frequencies (r 2 ), against genetic map distance (cM) between all marker pairs in each of the 12 linkage groups (LG) of the maritime pine genome. r2 was determined with the GGT 2.0 program, from the polymorphism data for 186 unrelated trees of the Aquitaine population. The 0.1 critical level of r2 was determined after Robbins et al. (2011). J Exp Bot, 62:1831–1845. [file 1471-2164-15-171-S8.DOC]
