# Supplementary material for: Genome-wide distribution of genetic diversity and linkage disequilibrium in a mass-selected population of maritime pine
Source: BMC Genomics. 2014 Mar 1;15:171. doi: 10.1186/1471-2164-15-171 (PMC4029062; doi:10.1186/1471-2164-15-171)
Supplement: Additional file 9 — Distribution of long distance intra-chromosomal linkage disequilibrium (LD) as estimated by r 2 . This distribution was used as a null model to test the significance of inter-chromosomal LD potentially due epistatic selection. [file 1471-2164-15-171-S9.DOC]

**Additional File 9:** Distribution of long distance intrachromosomal linkage disequilibrium (LD) as estimated by r^2^. This distribution was used as a null model to test the significance of interchromosomal LD potentially due epistatic selection.
