# Supplementary material for: Genome-wide distribution of genetic diversity and linkage disequilibrium in a mass-selected population of maritime pine
Source: BMC Genomics. 2014 Mar 1;15:171. doi: 10.1186/1471-2164-15-171 (PMC4029062; doi:10.1186/1471-2164-15-171)
Supplement: Additional file 11 — Estimates of effective population sizes. [file 1471-2164-15-171-S11.DOC]

**Additional file 11**: Estimates of effective population sizes.

One of the parameters that affect accuracy of genomic selection is the effective population size (N_e_). Here we discussed three possible attempts made to predict N_e_.

🡪Theoretically N_e_ can be derived from the following equation: ρ=4N_e_r (also expressed as C=4N_e_c), where r is the per-generation recombination rate between adjacent sites in bp (estimates of r require knowledge of the ratio of genetic map distance (say 1800cM) to physical map distance (say 24Gb)), and ρ the population recombination rate per bp, that can be directly estimated from DNA samples as explained by Stumpf and McVean [1]. Because of ascertainment bias (combined with an unknown ascertainment scheme), ρ based on SNP polymorphism or estimated using approximate Bayesian methods from combined polymorphism and LD, cannot be reliably estimated from the data generated in this study. Based on the biased sample of the SNPs detected within the 248 contigs (i.e. 714 pair wise comparisons) we actually found a negative value for ρ (-0.0005141). The atypical LD pattern (also validated for varying MAF thresholds > 5, 10 or 20% and varying distances between sites < 300, 500, 1000bp) charted below is a clear illustration of this bias: recombination can only increase with the physical distance! As stated in the manuscript the strong bias in the selection of the SNPs precludes the use of this estimate of ρ for any evolutionary interpretation.

| 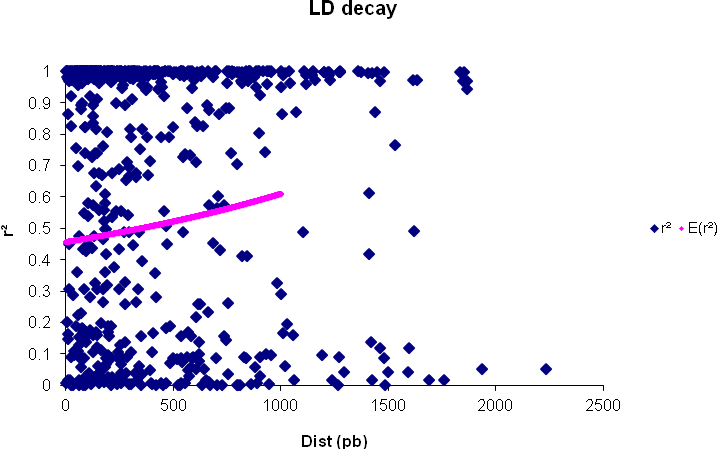 | Decay of linkage disequilibrium (r²) as a function of the distance between pairs of sites. The pink curve represents the expected values of r² [E(r²)] versus the distance between pairs of sites in bp. |
| --- | --- |

🡪 If neutral mutations are occurring at a locus at a rate μ per copy per generation, and each one occurs at a different site (the “infinite sites” model) and the effective population size is N_e_, it was showed that the expected number of nucleotide differences between two sequences, θπ, equals 4N_e_µ for a population in mutation drift-equilibrium [2]. With respect to µ, Willyard et al. [3] estimated it from 11 genes in four pine species representing pine major lineages and found that it ranged from 0.7 to 1.31*10^-9^/bp/year. Considering an average generation time of about 10 years this would translate to 0.7-1.31*10^-8^/bp/generation. With respect to θπ, an average estimate of 3.2*10^-3^ was provided by Lepoittevin [4] for silent mutations in the Landes population based on amplicon resequencing in 25 genes (unpublished data). Similar estimates were also provided [5,6] on a more limited set of genes based on a SNP discovery panel covering the whole natural range of maritime pine. All together these literature data can provide a crude range of N_e_: 61k < N_e_ < 114k, which is consistent with previous estimates found in the literature for conifer species sharing similar life-history traits (outcrossing mating system, wind pollination, dominant position in ecosystem where they are spread over large territories), e.g. from 96k to 182k in *Picea* spp. [7] and 94k in *Pinus taeda* [3]. However, the validity of these predictions will have to be validated considering departure from the assumption of mutation drift-equilbrium, because maritime pine populations have most probably undergone bottlenecks especially in the Northern part of its distribution.

🡪 N_e_ can also be estimated based on inbreeding coefficients [8]. It is important to state though that the Maritime Pine base population (FGB) consists of truly unrelated individuals. Calculation of the mean kinship value actually yielded a slightly negative value (-0.005) which means that any estimate of Ne based on relatedness will also be negative, although in reality it should be interpreted as representing very low relatedness and hence a large effective size.

References

1. Stumpf MP, McVean GA: **Estimating recombination rates from population genetic data**. *Nat Rev Genet, 2003,*  **4**:959-968.
2. Tajima, F.: **Evolutionary relationship of DNA sequences in finite populations**. *Genetics* 1983, **105**:437–460.
3. Willyard A, Syring J, Gernandt DS, Liston A, Cronn R: **Fossil calibration of molecular divergence infers a moderate mutation rate and recent radiations for *Pinus***. *Mol Biol Evol* 2007, **24**:90-101.
4. Lepoittevin C: **Association genetics in maritime pine (*Pinus pinaster* Ait.) for growth and wood quality traits**. PhD thesis, Bordeaux University; 2009.
5. Pot D, Mac Millan L, Echt C, le Provost G, Garnier-Géré P, Cato S, Plomion C: **Nucleotide diversity of genes involved in wood formation in Pinus pinaster and Pinus radiata**. *New Phytol* 2005, **167**:101-112.
6. Eveno E, Soto A, Gonzalez-Martinez S, Collada C, Guevara MA, Cervera MT, Léger P, Plomion C, Garnier-Géré P: **Contrasting outlier patterns on drought stress tolerance candidate genes in Pinus pinaster, as revealed by genetic differentiation analyses**. *Mol Biol Evol* 2008, **25**:417–437.
7. Bouille M, Bousquet J: Trans**-species shared polymorphisms at orthologous nuclear gene loci among distant species in the conifer *Picea* (Pinaceae): implications for the long-term maintenance of genetic diversity in trees.** *Am J Bot* 2005, **92**:63-73.
8. Caballero A, Toro MA: **Interrelations between effective population size and other pedigree tools for the management of conserved populations**. *Genet Res*, 2000, **75**:331–43.
