# Supplementary material for: Genome-wide distribution of genetic diversity and linkage disequilibrium in a mass-selected population of maritime pine
Source: BMC Genomics. 2014 Mar 1;15:171. doi: 10.1186/1471-2164-15-171 (PMC4029062; doi:10.1186/1471-2164-15-171)
Supplement: Additional file 12 — Description of the three component maps from Chancerel et al . (2013). [file 1471-2164-15-171-S12.DOC]

**Additional File 12**: Description of the three component maps from Chancerel *et al*. (2013).

| Map ID | **G2F** | **G2M** | **F2** |
| --- | --- | --- | --- |
| Number of framework markers | 550 | 619 | 1,121 |
| Map length (cM) | 1,447 | 1,425 | 1,708 |
