# Supplementary material for: Genome-wide distribution of genetic diversity and linkage disequilibrium in a mass-selected population of maritime pine
Source: BMC Genomics. 2014 Mar 1;15:171. doi: 10.1186/1471-2164-15-171 (PMC4029062; doi:10.1186/1471-2164-15-171)
Supplement: Additional file 13 — Geographic origin of the G0 trees. [file 1471-2164-15-171-S13.PDF]

GO ID on the database	ID used in this study	department	district	city	LATITUDE	LONGITUDE	forest stand	reference number in the map below
0001	1	40	Parentis en Born	Sainte Eulalie	44.273	-1.1837	Série5,div3	1
0003	3	40	Parentis en Born	Sainte Eulalie	44.273	-1.1837	Série5,div14,parc.2	1
0004	4	40	Parentis en Born	Sainte Eulalie	44.273	-1.1837	Série5,div14,parc.2	1
0005	5	40	Parentis en Born	Sainte Eulalie	44.273	-1.1837	Série4,div1,parc.2	1
0006	6	40	Parentis en Born	Sainte Eulalie	44.273	-1.1837	Série4,div1,parc.3	1
0008	8	40	Parentis en Born	Sainte Eulalie	44.273	-1.1837	Série4,div7,parc.2	1
0010	10	40	Parentis en Born	Sainte Eulalie	44.273	-1.1837	Série4,div7,parc.4	1
0011	11	40	Parentis en Born	Sainte Eulalie	44.273	-1.1837	Série3,div14,parc.1	1
0013	13	40	Parentis en Born	Sainte Eulalie	44.273	-1.1837	Série3,div5,parc.1	1
0014	14	40	Parentis en Born	Sainte Eulalie	44.273	-1.1837	Série3,div5,parc.3	1
0015	15	40	Parentis en Born	Sainte Eulalie	44.273	-1.1837	Série3,div7	1
0016	16	40	Parentis en Born	Sainte Eulalie	44.273	-1.1837	Série3,div7,parc2	1
0018	18	40	Parentis en Born	Sainte Eulalie	44.273	-1.1837	Série4,div6,parc.1	1
0019	19	40	Parentis en Born	Sainte Eulalie	44.273	-1.1837	Série4,div18,parc.4	1
0020	20	40	Parentis en Born	Sainte Eulalie	44.273	-1.1837	Série3,div6,parc.1	1
0023	23	40	Parentis en Born	Sainte Eulalie	44.273	-1.1837	Série3,div6,parc.3	1
0024	24	40	Parentis en Born	Ychoux	44.3288	-0.9633	Signal de Meyssonnave	2
0033	33	40	Parentis en Born	Ychoux	44.3288	-0.9633	Janoy Douks	2
0034	34	40	Parentis en Born	Ychoux	44.3288	-0.9633	Janoy Douks	2
0040	40	40	Parentis en Born	Parentis	44.3489	-1.0726	Etang	3
0052	52	40	Parentis en Born	Sainte Eulalie	44.273	-1.1837	Falot	1
0056	56	40	Parentis en Born	Parentis	44.3489	-1.0726	La Calle	3
0058	58	40	Parentis en Born	Parentis	44.3489	-1.0726	Dupouy	3
0059	59	40	Parentis en Born	Parentis	44.3489	-1.0726	Dupouy	3
0060	60	40	Parentis en Born	Parentis	44.3489	-1.0726	Dupouy	3
0061	61	40	Parentis en Born	Parentis	44.3489	-1.0726	Dupouy	3
0062	62	40	Parentis en Born	Parentis	44.3489	-1.0726	Dupouy	3
0063	63	40	Parentis en Born	Parentis	44.3489	-1.0726	Dupouy	3
0065	65	40	Parentis en Born	Parentis	44.3489	-1.0726	Ombreyres	3
0067	67	40	Parentis en Born	Parentis	44.3489	-1.0726	Lahitte	3
0068	68	40	Parentis en Born	Parentis	44.3489	-1.0726	Aoüqueyres	3
0070	70	40	Parentis en Born	Parentis	44.3489	-1.0726	Aoüqueyres	3
0072	72	40	Parentis en Born	Parentis	44.3489	-1.0726	Esleys	3
0077	77	40	Parentis en Born	Parentis	44.3489	-1.0726	Lahitte	3
0086	86	40	Parentis en Born	Sanguinet	44.4824	-1.0767	Lagrave	4
0101	101	40	Morcenx	Arengosse	44.0049	-0.7891	Dom. de Castillon	5
0103	103	40	Morcenx	Arengosse	44.0049	-0.7891	Dom. de Castillon	5
0105	105	40	Morcenx	Arengosse	44.0049	-0.7891	Dom. de Castillon	5
0106	106	40	Morcenx	Arengosse	44.0049	-0.7891	Dom. de Castillon	5
0108	108	40	Morcenx	Arengosse	44.0049	-0.7891	Dom. de Castillon	5
0110	110	40	Morcenx	Arengosse	44.0049	-0.7891	Dom. de Castillon	5
0111	111	40	Morcenx	Arengosse	44.0049	-0.7891	Dom. de Castillon	5
0115	115	40	Morcenx	Arengosse	44.0049	-0.7891	Dom. de Castillon(Pilh)	5
0116	116	40	Morcenx	Arengosse	44.0049	-0.7891	Dom. de Castillon	5
0117	117	40	Morcenx	Arengosse	44.0049	-0.7891	Dom. de Castillon	5
0121	121	40	Morcenx	Arengosse	44.0049	-0.7891	Dom. de Castillon	5
0122	122	40	Morcenx	Arengosse	44.0049	-0.7891	Dom. de Castillon	5
0123	123	40	Morcenx	Arengosse	44.0049	-0.7891	Dom. de Castillon	5
0124	124	40	Morcenx	Arengosse	44.0049	-0.7891	Dom. de Castillon	5
0125	125	40	Morcenx	Arengosse	44.0049	-0.7891	Dom. de Castillon(Bezin)	5
0128	128	40	Morcenx	Arengosse	44.0049	-0.7891	Dom. de Castillon	5
0131	131	40	Morcenx	Arengosse	44.0049	-0.7891	Dom. de Castillon	5
0133	133	40	Morcenx	Arengosse	44.0049	-0.7891	Dom. de Castillon	5
0134	134	40	Morcenx	Arengosse	44.0049	-0.7891	Dom. de Castillon	5
0137	137	40	Morcenx	Arengosse	44.0049	-0.7891	Dom. de Castillon	5
0138	138	40	Morcenx	Arengosse	44.0049	-0.7891	Dom. de Castillon	5
0142	142	40	Morcenx	Arengosse	44.0049	-0.7891	Dom. de Castillon	5
0146	146	40	Morcenx	Arengosse	44.0049	-0.7891	Dom. de Castillon	5
0147	147	40	Morcenx	Arengosse	44.0049	-0.7891	Dom. de Castillon	5
0150	150	40	Morcenx	Arengosse	44.0049	-0.7891	Dom. de Castillon	5
0152	152	40	Morcenx	Arengosse	44.0049	-0.7891	n. de Castillon(Pont de la Ma	5
0158	158	40	Morcenx	Arengosse	44.0049	-0.7891	Dom. de Castillon	5
0159	159	40	Morcenx	Arengosse	44.0049	-0.7891	Dom. de Castillon(Barnac)	5
0161	161	40	Morcenx	Onesse-Laharie	44.0487	-1.0052	ferme Poutchic	6
0205	205	33	Audenge	Ares	44.7672	-1.1404	Dom. Saussouze,parc. 25b	7
0212	212	33	Audenge	Ares	44.7672	-1.1404	Dom. Saussouze,parc. 24b	7
0217	217	33	Audenge	Ares	44.7672	-1.1404	Dom. Saussouze,parc. 24b	7
0221	221	33	Audenge	Ares	44.7672	-1.1404	Dom. Saussouze,parc. 23	7
0222	222	33	Audenge	Ares	44.7672	-1.1404	Dom. Saussouze,parc. 23	7
0235	235	33	Audenge	Ares	44.7672	-1.1404	Dom. Saussouze,parc. 23	7
0239	239	33	Audenge	Ares	44.7672	-1.1404	Dom. Saussouze,parc. 18b	7
0243	243	33	Audenge	Ares	44.7672	-1.1404	Dom. Saussouze,parc. 18b	7
0247	247	33	Audenge	Ares	44.7672	-1.1404	Dom. Saussouze,parc. 34b	7
0253	253	33	Audenge	Ares	44.7672	-1.1404	Dom. Saussouze,parc. 36	7
0281	281	33	Audenge	Lège	44.795	-1.147	sanatorium	8

0282	282	33	Audenge	Croix D'Hins	44.71	-0.8167	S.F.S.O.,parc.2	9
0283	283	33	Audenge	Lège-cap-ferret	44.7352	-1.2365	Série3,div16	10
0284	284	33	Audenge	Marcheprime	44.6915	-0.8541	Dom. de Marcheprime	11
0286	286	33	Audenge	Marcheprime	44.6915	-0.8541	om. de Marcheprime,parc.b:	11
0601	601	33	Belin	Hostens	44.4928	-0.6396	Joué	12
0602	602	33	Belin	Salles	44.5522	-0.8699	Lavignolle de Salles	13
1301	1301	33	Castelnau	Lacanau	44.9779	-1.0754	Dom.de Méjos(Saussouze)	14
1303	1303	33	Castelnau	Salaunes	44.9367	-0.8299	parc.337p,AT Lagueyte	15
1305	1305	33	Castelnau	Porge	44.873	-1.0929	Série2,div13	16
1306	1306	33	Castelnau	Porge	44.873	-1.0929	Série2,div12,pare-feu 69	16
1309	1309	33	Castelnau	Saumos	44.9214	-0.9881	Le Grand Bos	17
1311	1311	33	Castelnau	Le Temple	44.8791	-0.9908	Crasteneuve	18
1312	1312	33	Castelnau	Le Temple	44.8791	-0.9908	Crasteneuve	18
1313	1313	33	Castelnau	Le Temple	44.8791	-0.9908	Grande Craste	18
1317	1317	33	Castelnau	Le Temple	44.8791	-0.9908	NA	18
1319	1319	33	Castelnau	Le Temple	44.8791	-0.9908	route de Beagou	18
1320	1320	33	Castelnau	Le Temple	44.8791	-0.9908	NA	18
1322	1322	33	Castelnau	Sainte-Hélène	44.9646	-0.8849	NA	19
1324	1324	33	Castelnau	Saumos	44.9214	-0.9881	Sérigas	20
1325	1325	33	Castelnau	Saumos	44.9214	-0.9881	Le Moutin	20
1326	1326	33	Castelnau	Saumos	44.9214	-0.9881	Sautujanes	20
1327	1327	33	Castelnau	Saumos	44.9214	-0.9881	Sautujanes	20
1328	1328	33	Castelnau	Saumos	44.9214	-0.9881	Sautujanes	20
1329	1329	33	Castelnau	Saumos	44.9214	-0.9881	Sautujanes	20
1330	1330	33	Castelnau	Saumos	44.9214	-0.9881	Sautujanes	20
1331	1331	33	Castelnau	Saumos	44.9214	-0.9881	Sautujanes	20
1332	1332	33	Castelnau	Saumos	44.9214	-0.9881	Eyronnet	20
1333	1333	33	Castelnau	Saumos	44.9214	-0.9881	Eyronnet	20
1334	1334	33	Castelnau	Saumos	44.9214	-0.9881	Eyronnet	20
1335	1335	33	Castelnau	Saumos	44.9214	-0.9881	Eyronnet	20
1337	1337	33	Castelnau	Saumos	44.9214	-0.9881	Sautujanes	20
1338	1338	33	Castelnau	Porge	44.873	-1.0929	NA	16
1341	1341	33	Castelnau	Saumos	44.9214	-0.9881	SérieA,div12,Cratiou-Croutat	20
1342	1342	33	Castelnau	Saumos	44.9214	-0.9881	SérieA,div12,Cratiou-Croutat	20
1343	1343	33	Castelnau	Saumos	44.9214	-0.9881	SérieA,div12,Cratiou-Croutat	20
1344	1344	33	Castelnau	Saumos	44.9214	-0.9881	SérieA,div12,Cratiou-Croutat	20
1347	1347	33	Castelnau	Saumos	44.9214	-0.9881	Sautujanes	20
1350	1350	33	Castelnau	Brach	45.041	-0.9371	Mayne-Bernard	21
1352	1352	33	Castelnau	Brach	45.041	-0.9371	Mayne-Bernard	21
1354	1354	33	Castelnau	Brach	45.041	-0.9371	Mayne-Bernard	21
1356	1356	33	Castelnau	Saumos	44.9214	-0.9881	Gardillots	20
1902	1902	33	Lesparre	Naujac-sur-Mer	45.2536	-1.0257	Dom. du Flamand	22
1903	1903	33	Lesparre	Naujac-sur-Mer	45.2536	-1.0257	Dom. du Flamand	22
1904	1904	33	Lesparre	Vandays-Montalivet	45.355	-1.0603	Série1,Cap du Prat	23
2804	2804	33	St Laurent	Hourtin	45.1857	-1.071	série2,div11	24
3102	3102	33	St Symphorien	Hostens	44.4928	-0.6396	Les Cazalas	25
3105	3105	33	St Symphorien	Hostens	44.4928	-0.6396	Les Cazalas	25
3107	3107	33	St Symphorien	Hostens	44.4928	-0.6396	Les Cazalas	25
3108	3108	33	St Symphorien	Hostens	44.4928	-0.6396	NA	25
3110	3110	33	St Symphorien	Hostens	44.4928	-0.6396	NA	25
3111	3111	33	St Symphorien	Hostens	44.4928	-0.6396	NA	25
3112	3112	33	St Symphorien	Hostens	44.4928	-0.6396	Le Nets	25
3114	3114	33	St Symphorien	Hostens	44.4928	-0.6396	Berthos	25
3115	3115	33	St Symphorien	Hostens	44.4928	-0.6396	Les Anoudeys	25
3601	3601	33	Villandraut	Préchac	44.3988	-0.3534	Route Préchac à Cazalis	26
3602	3602	33	Villandraut	Préchac	44.3988	-0.3534	La Ribère(route de Lucmau)	26
3603	3603	33	Villandraut	Préchac	44.3988	-0.3534	Au Pesquey	26
3604	3604	33	Villandraut	Préchac	44.3988	-0.3534	Mansanqual	26
3801	3801	40	Castets	Léon	43.8842	-1.303	Dune de Huchet	27
3803	3803	40	Castets	Lit et Mixe	44.0328	-1.2577	Tuc de la Damiselle	28
3805	3805	40	Castets	Lit et Mixe	44.0328	-1.2577	Lestemguats	28
3806	3806	40	Castets	Lit et Mixe	44.0328	-1.2577	Tuc de la Damiselle (Petrocq	28
3808	3808	40	Castets	Lit et Mixe	44.0328	-1.2577	Lestinguats	28
3810	3810	40	Castets	Lit et Mixe	44.0328	-1.2577	oie ferrée,chemin de Miquel	28
3811	3811	40	Castets	Lit et Mixe	44.0328	-1.2577	Lestourneau	28
3812	3812	40	Castets	Lit et Mixe	44.0328	-1.2577	Haou de Gaillat	28
3813	3813	40	Castets	Lit et Mixe	44.0328	-1.2577	Mourens	28
3815	3815	40	Castets	Lit et Mixe	44.0328	-1.2577	Haou de Gaillat	28
3816	3816	40	Castets	Lit et Mixe	44.0328	-1.2577	Cabenious	28
3821	3821	40	Castets	Lit et Mixe	44.0328	-1.2577	La Fabrique	28
3825	3825	40	Castets	Lit et Mixe	44.0328	-1.2577	route de Miquéou à Naboud	28
3829	3829	40	Castets	Lit et Mixe	44.0328	-1.2577	Robin	28
3832	3832	40	Castets	Saint Julien en Born	44.0618	-1.226	Couilloy	29
3834	3834	40	Castets	Saint Julien en Born	44.0618	-1.226	Route d'Uza	29
3835	3835	40	Castets	Saint Julien en Born	44.0618	-1.226	Route St Julien-Uza	29
3837	3837	40	Castets	Saint Julien en Born	44.0618	-1.226	Le Mène	29
3838	3838	40	Castets	Saint Julien en Born	44.0618	-1.226	Le Mène	29
3839	3839	40	Castets	Saint Julien en Born	44.0618	-1.226	Baylet	29

3841	3841	40	Castets	Saint Julien en Born	44.0618	-1.226	Bellevue	29
3842	3842	40	Castets	Saint Julien en Born	44.0618	-1.226	Bue de Salvat	29
3843	3843	40	Castets	Saint Julien en Born	44.0618	-1.226	Orvignac	29
3845	3845	40	Castets	Saint Julien en Born	44.0618	-1.226	Orvignac	29
3846	3846	40	Castets	Saint Julien en Born	44.0618	-1.226	Route de Bias	29
4302	4302	40	Mimizan	Aureilhan	44.215	-1.1932	oie ferrée,160m station du la	30
4303	4303	40	Mimizan	Saint Julien en Born	44.0618	-1.226	NA	29
4304	4304	40	Mimizan	Saint Julien en Born	44.0618	-1.226	Leych(route St Paul-Mézos)	29
4306	4306	40	Mimizan	Mézos	44.077	-1.1678	Petre	31
4311	4311	40	Mimizan	Mézos	44.077	-1.1678	Embranchement(route Ones	31
4313	4313	40	Mimizan	Mézos	44.077	-1.1678	Fontaine de Ourlosse	31
4322	4322	40	Mimizan	Mézos	44.077	-1.1678	Maboude	31
4323	4323	40	Mimizan	Mézos	44.077	-1.1678	Maboude	31
4324	4324	40	Mimizan	Mézos	44.077	-1.1678	la Lande	31
4326	4326	40	Mimizan	Mézos	44.077	-1.1678	Maboude	31
4328	4328	40	Mimizan	Pontex les Forges	44.2412	-1.1211	Saint Trosse	32
4333	4333	40	Mimizan	Pontex les Forges	44.2412	-1.1211	Pélic	32
4334	4334	40	Mimizan	Saint Julien en Born	44.0618	-1.226	Lerte(route de Leych)	29
4702	4702	40	Roquefort	Retjons	44.1018	-0.2946	Saula de Mey	33
5101	5102	40	St Vincent de Tyrosse	Seignosse	43.69	-1.3751	La Montagne	34
5302	5302	40	Soustons	Magescq	43.7811	-1.2169	Route Mageescq-Dax	35
5303	5303	40	Soustons	Magescq	43.7811	-1.2169	série1, div9	35
5305	5305	40	Soustons	Moliets	43.8488	-1.3591	iv.6,Génicore,M.F. du Hourc	36
5307	5307	40	Soustons	Saint Girons	43.9551	-1.3052	div1,parc.3	37
5308	5308	40	Soustons	Saint Girons	43.9551	-1.3052	Mathiouicq	37
5310	5310	40	Soustons	Seignosse	43.69	-1.3751	La Montagne	34
5311	5311	40	Soustons	Soustons	43.7557	-1.3262	NA	38
7101	7101	17	Charentes Maritimes	de la Coubre	45.6923	-1.1836	Série1,div7,parc.31	39
7104	7104	17	Charentes Maritimes	Arvert	45.7416	-1.1274	Les Usages	40
8371	8371	33	Saint Jean d'Illac	Saint Jean d'Illac	44.8112	-0.7822	NA	41
8374	8374	33	Saint Jean d'Illac	Saint Jean d'Illac	44.8112	-0.7822	NA	41
13T14	13T14	33	Castelnau	Le Temple	44.8791	-0.9908	Bourdieu	18
13T16	13T16	33	Castelnau	Le Temple	44.8791	-0.9908	Bourdieu	18

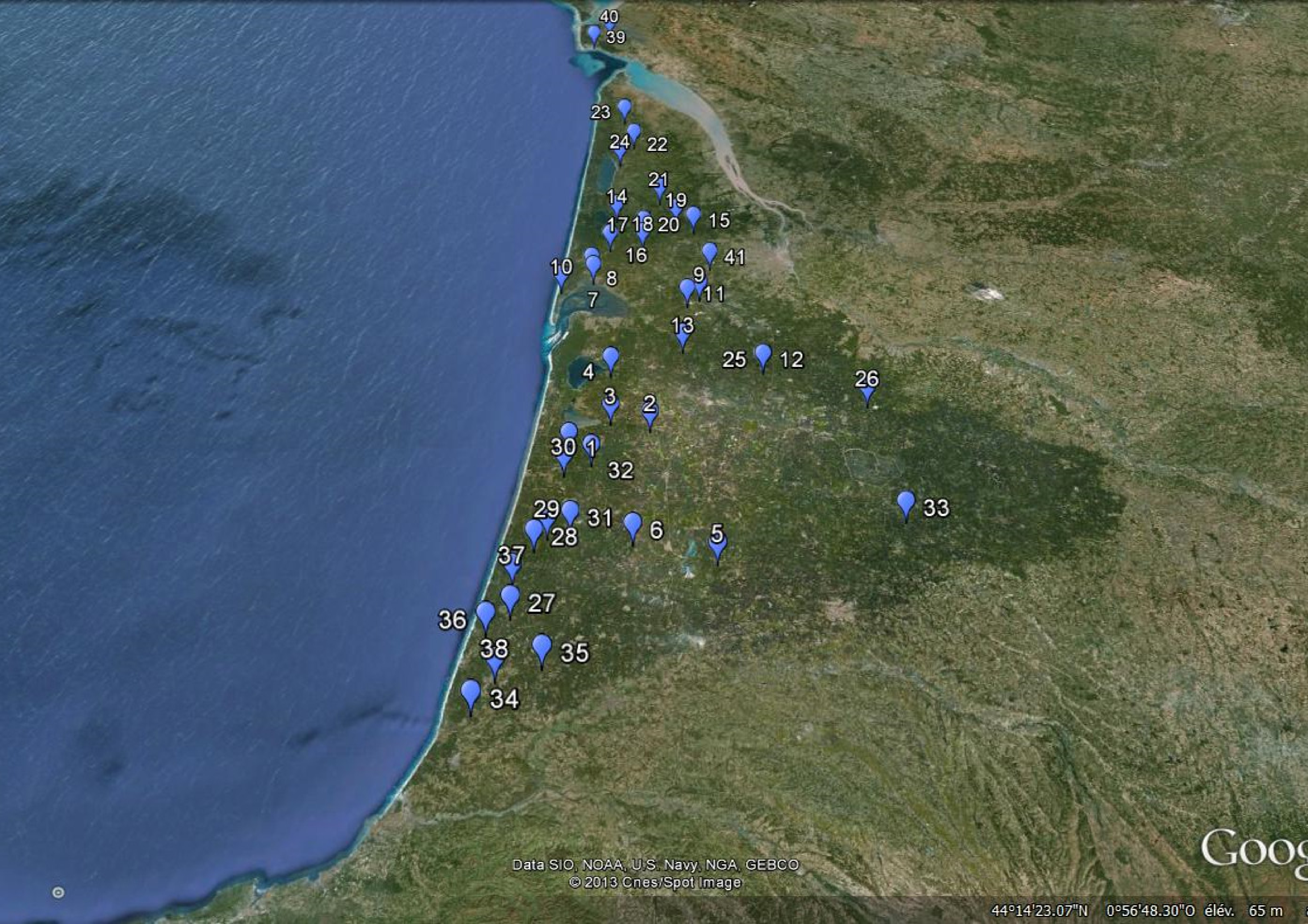
40
39
23
24
22
21
14
19
15
17
18
20
16
41
10
8
9
11
7
13
25
12
4
26
3
2
30
1
32
29
31
33
6
28
5
37
27
36
38
35
34
Data SIO, NOAA, U.S. Navy, NGA, GEBCO
© 2013 Cnes/Spot Image
44°14'23.07"N 0°56'48.30"O élév. 65 m
