# Supplementary material for: Genome-wide distribution of genetic diversity and linkage disequilibrium in a mass-selected population of maritime pine
Source: BMC Genomics. 2014 Mar 1;15:171. doi: 10.1186/1471-2164-15-171 (PMC4029062; doi:10.1186/1471-2164-15-171)
Supplement: Additional file 15 — Bandwidth values (cM) obtained by kernel density analysis for the composite linkage map obtained with LPmerge. [file 1471-2164-15-171-S15.DOC]

**Additional File 15**: Bandwidth values (cM) obtained by kernel density analysis for the composite linkage map obtained with LPmerge.

| Linkage groups | Bandwidth (cM) |
| --- | --- |
| LG1 | 5.5 |
| LG2 | 4.4 |
| LG3 | 6.5 |
| LG4 | 7 |
| LG5 | 7.6 |
| LG6 | 7.1 |
| LG7 | 9.4 |
| LG8 | 5.5 |
| LG9 | 6.4 |
| LG10 | 13.3 |
| LG11 | 6.2 |
| LG12 | 5.6 |
